# Eliciting the silent lucensomycin biosynthetic pathway in *Streptomyces cyanogenus* S136 via manipulation of the global regulatory gene *adpA*

**DOI:** 10.1038/s41598-021-82934-6

**Published:** 2021-02-10

**Authors:** Oleksandr Yushchuk, Iryna Ostash, Eva Mösker, Iryna Vlasiuk, Maksym Deneka, Christian Rückert, Tobias Busche, Victor Fedorenko, Jörn Kalinowski, Roderich D. Süssmuth, Bohdan Ostash

**Affiliations:** 1 Department of Genetics and Biotechnology, Ivan Franko National University of Lviv, 4 Hrushevskoho st., Rm. 102, Lviv, 79005 Ukraine; 2 Institut für Chemie, Technische Universität Berlin, Straße des 17. Juni 124, 10623 Berlin, Germany; 3 Technology Platform Genomics, CeBiTec, Bielefeld University, Universitätsstraße 27, 33615 Bielefeld, Germany

**Keywords:** Microbiology, Antimicrobials, Microbial genetics

## Abstract

Actinobacteria are among the most prolific sources of medically and agriculturally important compounds, derived from their biosynthetic gene clusters (BGCs) for specialized (secondary) pathways of metabolism. Genomics witnesses that the majority of actinobacterial BGCs are silent, most likely due to their low or zero transcription. Much effort is put into the search for approaches towards activation of silent BGCs, as this is believed to revitalize the discovery of novel natural products. We hypothesized that the global transcriptional factor AdpA, due to its highly degenerate operator sequence, could be used to upregulate the expression of silent BGCs. Using *Streptomyces cyanogenus* S136 as a test case, we showed that plasmids expressing either full-length *adpA* or its DNA-binding domain led to significant changes in the metabolome. These were evident as changes in the accumulation of colored compounds, bioactivity, as well as the emergence of a new pattern of secondary metabolites as revealed by HPLC-ESI-mass spectrometry. We further focused on the most abundant secondary metabolite and identified it as the polyene antibiotic lucensomycin. Finally, we uncovered the entire gene cluster for lucensomycin biosynthesis (*lcm*), that remained elusive for five decades until now, and outlined an evidence-based scenario for its *adpA*-mediated activation.

## Introduction

Over decades, prokaryotes were one of the most prolific sources of antibiotics, rivaled only by lower fungi^1^. Among antibiotic-producing prokaryotes, the classes Actinobacteria and Myxobacteria currently dominate^2,3^, although exploration of the other taxa could change this view significantly in the near future^4–6^*.* The actinobacterial genus *Streptomyces* probably still is the most successful provider of drugs for healthcare and agriculture^1^. Beginning with seminal works of Selman Waksman^7,8^, the majority of known antibiotic compounds from *Streptomyces* were isolated through classical screening approaches. However, due to high rediscovery rates, this approach has gradually lost its efficiency^9^. More recently, the sequencing of *Streptomyces* spp. genomes revealed a large hidden potential for the production of antibiotics and other secondary metabolites. Exemplary studies of the fully sequenced genomes of *S. coelicolor* and *S. avermitilis* revealed many more biosynthetic gene clusters (BGCs) than compounds previously known to be produced by these species^10,11^. Ever since, each new sequenced *Streptomyces* genome gave rise to the idea that most of the actinobacterial BGCs are silent, i.e. that they are not associated with the production of certain compounds under typical cultivation conditions, most likely due to low or zero transcription^12,13^.

Today’s relative simplicity of in silico identification of BGCs within genomic sequences^14,15^ prompted the search for approaches to activate silent BGCs. Certain mutations in genes for ribosomal proteins and RNA polymerases are capable of eliciting the expression of the silent secondary metabolome^16,17^. Extracellular triggers, like xenobiotics, rare earth elements or co-cultivation with other bacteria were also shown to activate the production of novel antibiotics by streptomycetes^18–22^. Manipulations of either regulatory elements within the putative BGCs^23,24^, or BGC-situated regulatory genes^23,25,26^ often lead to the activation of silent BGCs. As antibiotic production in streptomycetes is tightly intertwined with morphogenesis, global regulators that coordinate both aforementioned processes can be useful tools for activation of silent BGCs for known or cryptic biosynthetic pathways^27–32^. Such an approach could be especially useful for activation of the BGCs lacking cluster-situated regulatory genes.

The AraC family transcriptional factor (TF) AdpA is a global regulator of secondary metabolism and morphogenesis, and was successfully used to activate secondary metabolism^29,30^. AdpA appears as an appealing tool in this regard because it binds to a highly degenerate operator sequence^33^ and thus possesses an enormous regulon^34^. The overexpression of such a promiscuous TF could enforce binding to numerous (probably unrecognizable by AdpA under physiological conditions) sites on the chromosome, impacting the expression of multiple genes and operons. The biosynthesis of many antibiotics in streptomycetes is AdpA-regulated and AdpA TFs are almost identical across different *Streptomyces* species and therefore likely display the same properties^35^. AdpA-binding sites are common in silent BGCs and overexpression of *adpA* genes could be used for high-throughput activation of such BGCs in strains of unknown genomic background.

In this work we used *Streptomyces cyanogenus* S136 as a proof-of-concept to probe the above conjecture. This strain is known to produce deeply colored angucyclines, polyketides from the landomycin family, in a medium-dependent fashion; furthermore, sequencing revealed the presence of > 30 BGCs in its genome. We show that the secondary metabolome of *S. cyanogenus* actively responds to the overexpression of heterologous *adpA* genes with the production of novel secondary metabolites. Here we focused on the genetic basis of the production of the most abundant compound, lucensomycin, known for its antifungal activity. From our results we deduced a scheme for AdpA-mediated regulation of lucensomycin biosynthesis. Finally, we explored the functional significance of different parts of the AdpA protein for its ability to induce lucensomycin production. Our results expand the current knowledge of the secondary metabolome of *S. cyanogenus* and offer new insights into the ways of activation of silent BGCs in *Streptomyces.*

## Results

### Genome of *Streptomyces cyanogenus* encodes sizable secondary metabolome that responds to *adpA*

In the genome of *S. cyanogenus* S136, in addition to the *lan* cluster coding for landomycin biosynthesis, at least 33 putative BGCs were found under the default antiSMASH search conditions. From these, 16 BGCs shared significant similarities to known BGCs (ESM Fig. S1). These were the BGCs for informatipeptin, pimaricin, naphtomycin, chlorothricin and different RiPPs, furthermore for terpenoids hopene, isorenieratene and albaflavenone, the siderophore desferrioxamine B, ectoine, melanins and the spore pigment of polyketide nature. It is worth mentioning that most of the antibiotic BGCs putatively encode the biosyntheses of polyketide compounds (e.g. pimaricin, naphtomycin, chlorothricin).

In *S. cyanogenus* S136, the gene *SCY4743* codes for a nonfunctional AdpA, and expression of heterologous *adpA* genes activated landomycin biosynthesis under conditions where S136 normally does not produce this antibiotic^36^. This observation prompted us to look deeper into the secondary metabolome of *S. cyanogenus* strains carrying heterologous versions of *adpA*. Initially, we chose a landomycin-nonproducing mutant ΔlanI7^37^ for these experiments. The mutant strain was chosen in order to eliminate the competition for precursors (acyl-CoA, carbohydrates) of the landomycin pathway with potentially activated BGCs directing the production of biogenetically related compounds. Plasmids pGM4181 and pGM4181d carrying full *adpA* of *S. albus* J1074 and its DNA-binding domain (DBD), respectively, under the control of *moeE5p* promoter, were introduced into *S. cyanogenus* ΔlanI7. This promoter allows strong constitutive gene expression in *S. cyanogenus*^36^*.*

**Figure 1:**
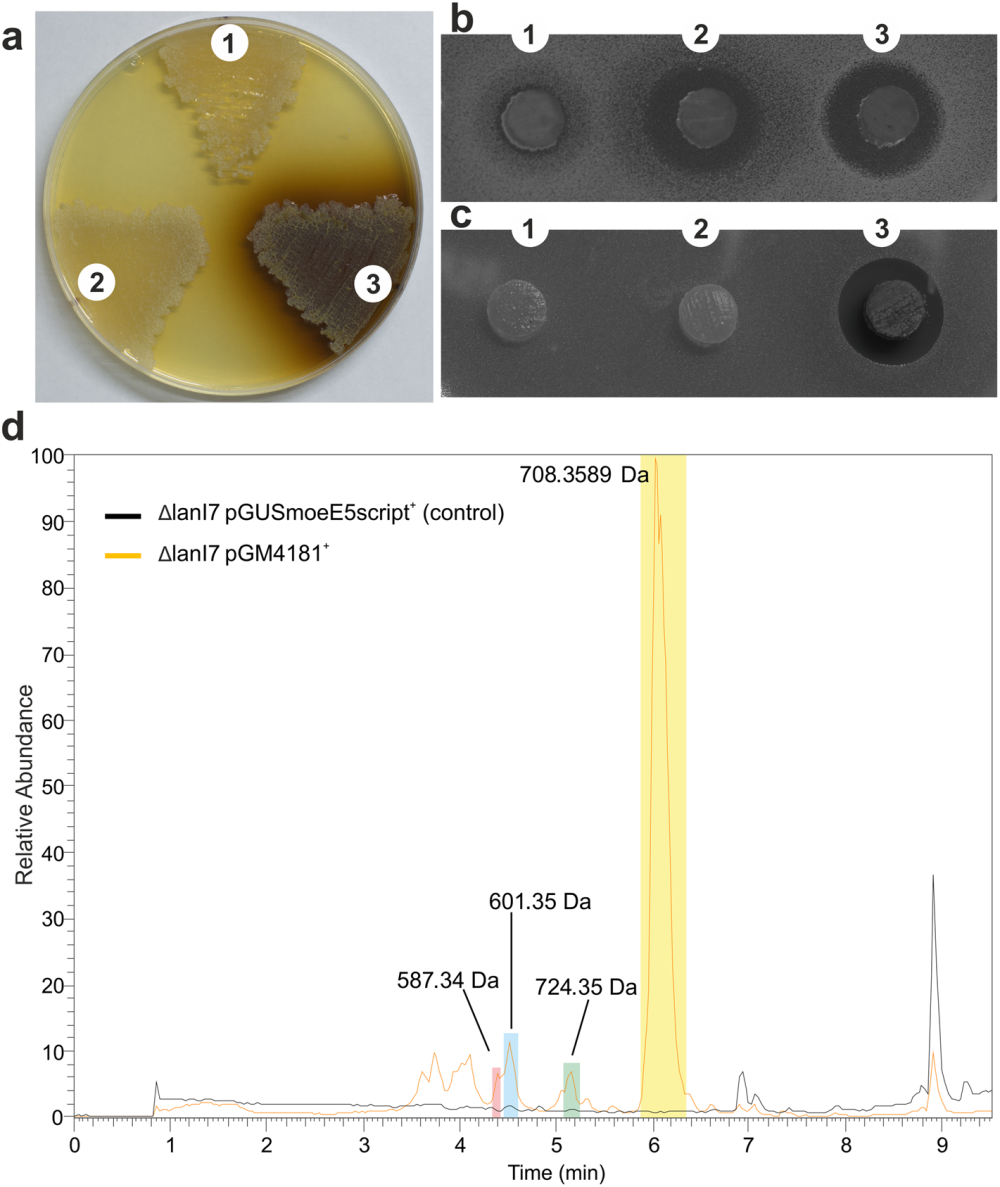
The *adpA* gene activates silent secondary metabolome of *S. cyanogenus*. (**a**) Phenotypes of *S. cyanogenus* ΔlanI7 (1), *S. cyanogenus* ΔlanI7 pGM4181d^+^ (2; DBD of *adpA*) and *S. cyanogenus* ΔlanI7 pGM4181^+^(3; full-length *adpA*) after 168 h of growth on TSB agar; *B. cereus* (**b**) and *D. hansenii* (**c**) growth inhibition assays using agar plugs taken from 120 h old ISP5 lawns of *S. cyanogenus* ΔlanI7 (1), *S. cyanogenus* ΔlanI7 pGM4181d^+^ (2) and *S. cyanogenus* ΔlanI7 pGM4181^+^(3). (**d**) LC–MS analysis of the secondary metabolites produced in liquid YMPG medium by *S. cyanogenus* ΔlanI7 pGUSmoeE5script^+^ (control) and *S. cyanogenus* ΔlanI7 pGM4181d^+^; four new mass peaks appeared in the chromatogram for the latter strain.

*S. cyanogenus* ΔlanI7 pGM4181^+^, but not pGM4181d^+^, started producing a dark brown compound when grown on TSA plates or in TSB media (Fig. 1a). However, we were not able to extract this compound from culture broths or solid agar media with common solvents. Likewise, mass spectrometry of the culture broth failed to identify distinct mass peaks. We suspected that the observed pigmentation was due to accumulation of tyrosine-based polymers, melanins, because in several *Streptomyces* species production of the latter is known to be upregulated by AdpA^38^. The structure elucidation of these polymers remains elusive due to their heterogeneity and poor solubility^39,40^. We therefore tentatively suggest that melanin-type compounds are accumulated in *S. cyanogenus* in presence of *adpA*. Indeed, its genome carries two *melC* homologs responsible for melanin biosynthesis, and their expression is activated upon pGM4181 introduction (ESM Fig. S2).

*S. cyanogenus* ΔlanI7 displayed weak activity against Gram-positive bacteria when grown on ISP5 agar. Such an activity was significantly increased in *S. cyanogenus* ΔlanI7 pGM4181^+^ and pGM4181d^+^ (Fig. 1b). Neither initial nor recombinant strains showed activity against *E. coli* (data not shown). The *adpA* expression in *S. cyanogenus* ΔlanI7 elicited strong antifungal activity, apparently absent in the control strain (Fig. 1c). Encouraged by this finding, we tested a number of agar media and found that many of them (but not all, see ESM Fig. S3) supported the production of antifungal compound(s) by *S. cyanogenus* ΔlanI7 pGM4181^+^ and pGM4181d^+^. We noted that for certain media the expression of DBD of AdpA is sufficient to induce the occurrence of antifungal activity (ESM Fig. S3), but the full-length gene was found to be more efficient. YMPG medium supported the highest level of antifungal activity, and was used for further analysis of *adpA* effects on the *S. cyanogenus* metabolome.

We grew ΔlanI7 and its pGM4181^+^ derivative in liquid YMPG medium and analyzed the biomass and supernatant extracts with LC–MS; the same was done in parallel for wild type (S136) and its pGM4181^+^ derivative. The peaks for landomycin A and its precursors dominated the chromatograms of the extracts from S136 and S136 pGM4181^+^; no qualitative differences were found between the HPLC chromatograms of these strains (ESM Fig. S4). In contrast, at least four new mass peaks occurred in the HPLC chromatograms of ΔlanI7 carrying pGM4181 (Fig. 1d). The *m/z* values corresponding to the less abundant mass peaks had no matches in the databases of natural compounds. The molecular mass of the most prominent “activated” ion peak (708.35 Da, [M + H]^+^) matched to that of the polyene antibiotic lucensomycin (Lcm)^41,42^, and thus we focused our attention on this peak. Lcm is known as antifungal compound, and its molecular mass peak is strongly prevailing in the HPLC–MS analytics of the metabolome of the ΔlanI7 pGM4181^+^ strain. We suppose therefore that Lcm is responsible for the antifungal activity exhibited by this strain. Nevertheless, we cannot exclude the contribution of the other, as yet unknown, compounds accumulated by ΔlanI7 pGM4181^+^ to antifungal activities of its extracts and agar plugs, although this contribution (if present) is likely insignificant.

### Optimized conditions for expression of antifungal activity in *S. cyanogenus* ΔlanI7

**Figure 2:**
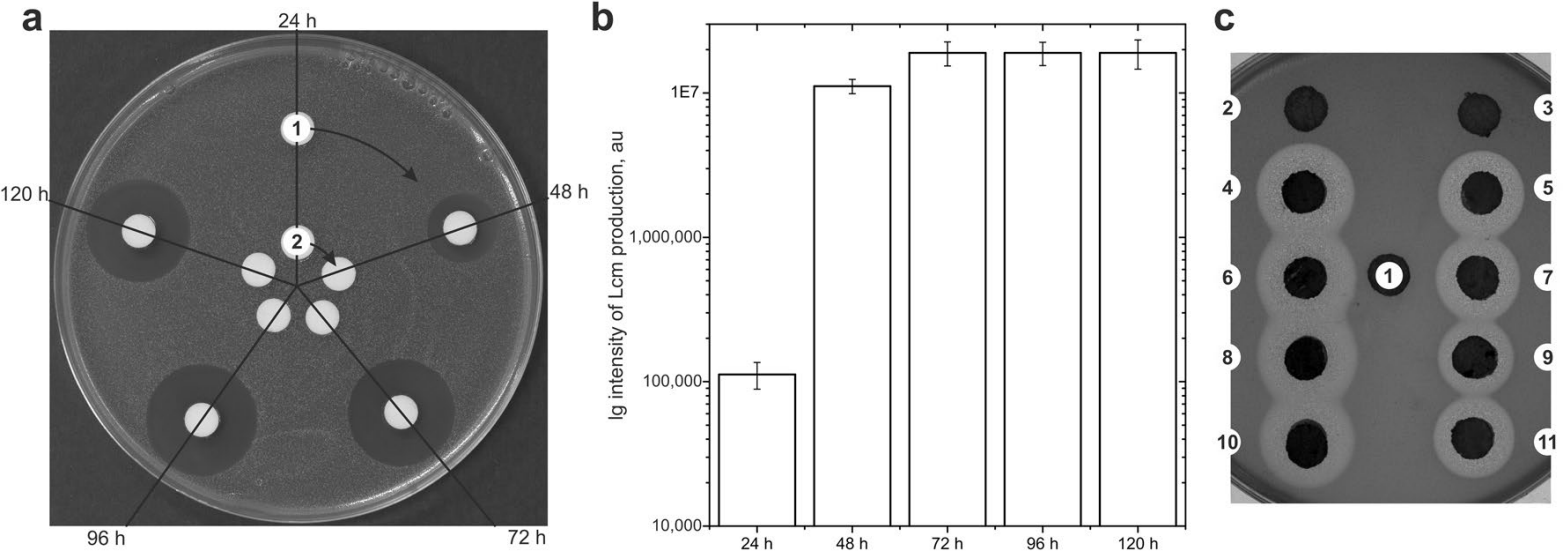
Antifungal activity of *S. cyanogenus* ΔlanI7 pGM4181^+^ as a function of cultivation time (**a**, **b**) and origin of *adpA* gene (**c**). For *D. hansenii* growth inhibition assay (**a**) the extracts were prepared from YMPG-grown ΔlanI7 pGM4181^+^ (1) and ΔlanI7 (2) at timepoints shown in the figure. The initial strain ΔlanI7 (2) showed no activity at all investigated time points. LC–MS quantification of the Lcm mass peak in the same extracts (**b**) was in agreement with the results of the bioassay. Data represent mean values of three independent experiments ± 2SD. (**c**) *D. hansenii* growth inhibition assay showing that *adpA* genes (and corresponding DNA-binding domains, DBD), other than *XNR_4181,* were able to activate Lcm production in *S. cyanogenus* ΔlanI7: (4,5)—ΔlanI7 pGMSCO^+^ and pGMSCOd^+^, overexpressing *S. coelicolor adpA* and its DBD, respectively; (6,7)—ΔlanI7 pGMSCLA^+^ and pGMSCLAd^+^, *S. clavuligerus adpA* and its DBD, respectively; (8,9)—ΔlanI7 pGM4181^+^ and pGM4181d^+^, *S. albus adpA* and its DBD, respectively; (10,11)—ΔlanI7 pOOB95d^+^ and pGMSGHd^+^, *S. ghanaensis adpA* and its DBD, respectively. Initial strain (1) as well as strains carrying native *adpA* from *S. cyanogenus* (ΔlanI7 pGMSCY^+^) and its DNA-binding domain (ΔlanI7 pGMSCYd^+^) do not inhibit growth of *D. hansenii.* Agar plugs were cut from the lawns grown on YMPG agar for 120 h.

We set out to identify the optimal conditions for the production of the antifungal compound by *S. cyanogenus* ΔlanI7 pGM4181^+^ prior to its detailed structural verification. In liquid YMPG the production of an antifungal compound became detectable after 48 h of growth and reached its peak at 96 h (Fig. 2a). The bioassay data correlated with the abundance of the 708.35 Da peak in HPLC chromatograms of the same extracts (Fig. 2b). No antifungal activity was observed in the extracts of ΔlanI7 biomass harvested at 24 h, 48 h, 72 h, 96 h and 120 h (Fig. 2a).

Subsequently, we introduced into *S. cyanogenus* ΔlanI7 a set of full-length *adpA* genes and corresponding DBDs—*adpAdbd—*from different *Streptomyces* species. This was carried out to determine whether and to what extent different *adpA* genes induce the antifungal activity. All heterologous *adpA* genes were shown to be strong inducers, and *adpAdbd*’s were less active in this regard (Fig. 2c). The overexpression of neither native *adpA* (*SCY4743*) nor its *adpAdbd* led to the activation of antifungal activity, once again pointing to the non-functionality of SCY4743 in *S. cyanogenus.*

### The AdpA-induced compound corresponds to the polyene antibiotic lucensomycin

Using the optimized production conditions, we were able to purify 40 mg of target antifungal compound from ΔlanI7 pGM4181^+^ grown in 2 L of YMPG (shaken flasks). In disk diffusion assay the compound showed activity only against fungal species (ESM Table S2, ESM Fig. S5). High-resolution mass spectrometric (MS) measurements (calculated for Lcm: 706.3444 Da ([M − H]^−^), 708.3590 Da ([M + H]^+^); observed: 706.3447 ([M − H]^−^) and 708.3586 ([M + H]^+^); mass tolerance ≤ 5 mmu) and the fragmentation pattern of the compound (ESM Fig. S6) are in full agreement with the structure of Lcm^42,43^.

### Description of the lucensomycin (*lcm*) BGC in *S. cyanogenus*

**Figure 3:**
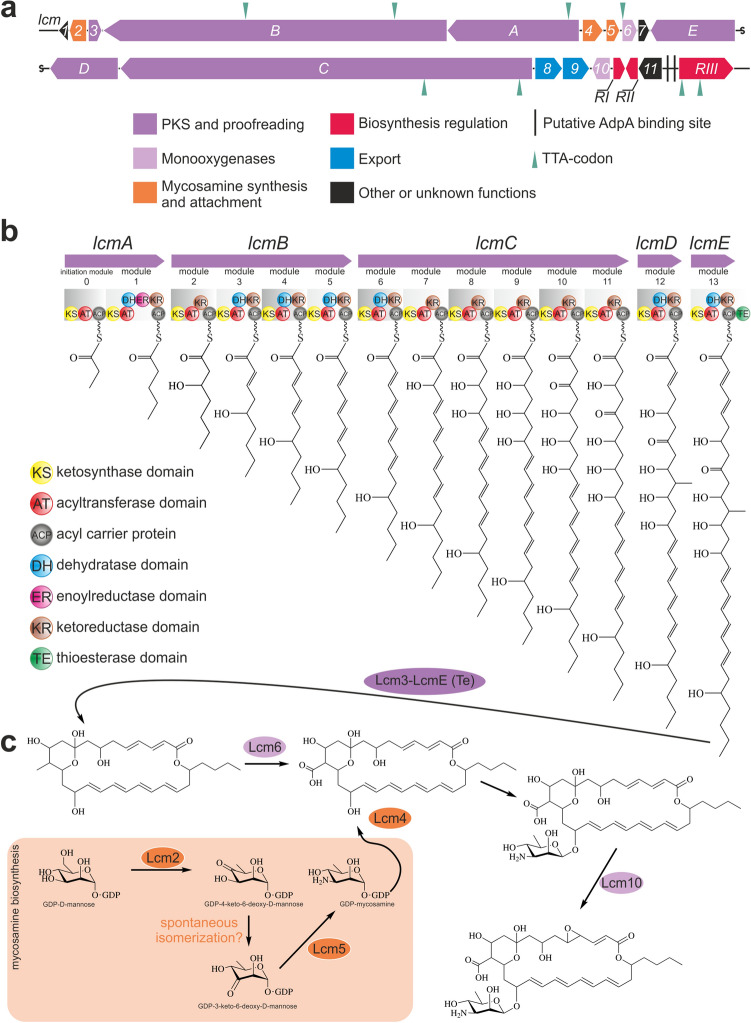
A scheme of Lcm biosynthetic gene cluster (*lcm* BGC) (**a**) and proposed sequence for the Lcm PKS assembly line (**b**) with the tailoring reactions (**c**). For more details, please see the main text and Table 1.

A survey of BGCs within the *S. cyanogenus* genome (see above) readily led us to recognize that one BGC shared significant similarity to the pimaricin (also known as natamycin) BGC (*pim* cluster)^44–46^. As Lcm and pimaricin are structural homologs^41^, we suggested that activation of this particular BGC results in Lcm accumulation by *S. cyanogenus* ΔlanI7 pGM4181^+^ and pGM4181d^+^. We therefore refer to this BGC as the *lcm* (**l**u**c**enso**m**ycin) BGC (accession number MW175861). Besides the *lcm* BGC, a few other genes for type I polyketide synthases were found within the genome of *S. cyanogenus* (ESM Fig. S1)*.* There was an overall synteny between the *lcm* BGC and the BGC already proposed to control Lcm production in *S. achromogenes* NRRL 3125^47^. However, the Lcm BGC from NRRL 3125 appeared to be incomplete (see below) and its nucleotide sequence is absent in publicly available databases; this precludes their detailed comparison. Nineteen ORFs were annotated within the *lcm* BGC (Table 1; Fig. 3a), among which 18 are similar to genes found in the other polyene BGCs (Table 1). We took advantage of detailed knowledge of the biosynthesis of pimaricin and structural similarities between the latter and Lcm to propose a tentative biochemical pathway leading to Lcm.

**Table 1:** Description of the ORFs discovered in *lcm* gene cluster.

*lcm*	The closest homologue from antibiotic BGCs	AA sequence identity(%)/E-value	Putative product	Putative function in Lcm biosynthesis
*lcm1*	N/A		–	–
*lcm2*	AviE3 (avilamycin BGC)	61.92/5e^−111^	GDP-D-mannose 4,6-dehydratase (COG1089)	Transformation of GDP-D-mannose into GDP-4-keto-6-deoxy-D-mannose
*lcm3*	FscTE (candicidin BGC)	60,08/5e^−70^	Type II thioesterase (COG3208)	Removal of aberrant intermediates
*lcmB*			PKS modules 2–5	Lcm aglycone assembly line
*lcmA*			PKS initiation and 1 modules	Lcm aglycone assembly line
*lcm4*	AmphDI (amphotericin B BGC)	58,89/4e^−156^	GDP-mycosamine glycosyltransferase (COG1819)	Glycosylation of Lcm aglycone with mycosamine
*lcm5*	FscMII (candicidin BGC)	74,43/3e^−154^	GDP-3-keto-6-deoxy-D-mannose C-3 aminotransferase (COG0399)	Transformation of GDP-3-keto-6-deoxy-D-mannose into GDP-mycosamine
*lcm6*	PimG (pimaricin BGC)	64,78/5e^−153^	Cytochrome P450 monooxygenase (COG2124)	Formation of carboxyl group at C-12
*lcm7*	RimH (rimocidin BGC)	66,13/8e^−13^	Ferredoxin (COG1141)	Electron transfer in P450 system
*lcmE*			PKS module 12	Lcm aglycone assembly line
*lcmD*			PKS module 11	Lcm aglycone assembly line
*lcmC*			PKS modules 5–10	Lcm aglycone assembly line
*lcm8*	PimA (pimaricin BGC)	57,71/3e^−147^	ABC transporter (COG1132)	Export of Lcm
*lcm9*	AmphG (amphotericin B BGC)	56,20/1e^−171^	ABC transporter (COG1132)	Export of Lcm
*lcm10*	NysL (nystatin BGC)	42,46/3e^−81^	Cytochrome P450 monooxygenase (COG2124)	Epoxidation of Lcm aglycone at C4-C5
*lcmRI*	NysRIV (nystatin BGC)	39,38/1e^−29^	HTH-LuxR domain containing TF	Regulation of Lcm biosynthesis
*lcmRII*	PimM(pimaricin BGC)	40,1/2e^−32^	HTH-LuxR domain containing TF	Regulation of Lcm biosynthesis
*lcm11*	PimE	79,34/0	Cholesterol oxidase (COG2303)	“Fungal sensor” for ergosterol detection
*lcmRIII*	PimR	77,9/0	Transcriptional regulator	Regulation of Lcm biosynthesis

*Assembly of Lcm aglycone.* There are five PKS genes within the *lcm* BGC, four of which (*lcmB, lcmC, lcmD* and *lcmE*) are colinear to *pimS1, pimS2, pimS3* and *pimS4*. It is therefore reasonable to propose analogous functions for *pim* and *lcm* homologs in the polyene aglycone assembly (Fig. 3b). However, the *lcmA* gene encodes for a PKS in which the initiation module is apparently fused to the first PKS module. This kind of organization is unprecedented in the biosynthesis of polyenes, although widely known for other type I PKS-derived products, such as erythromycin^48^. Most likely, this reflects the unique structure of Lcm featuring a *n*-propyl side chain. From analysis of the LcmA domain order, we propose that KS_0_ would activate methylmalonyl-CoA as the starter substrate, and the second module (featuring full reductive loop) would incorporate malonyl-CoA, thus leading to a fully reduced C5 unit. Indeed, by a number of in silico tools KS_0_ and KS_1_ are reliably predicted to bind methylmalonyl- and malonyl-CoA, respectively, lending support for our conjecture. The identified type II thioesterase Lcm3, a homolog of FscTE from the biosynthesis of polyene candicidin^49^, would be assigned responsible for the proofreading in Lcm aglycone formation.

*Post-PKS modifications of Lcm aglycone*. This is achieved in three sequential tailoring reactions. First, a carboxy group is installed at position C-12 (Fig. 3), most probably via action of monooxygenase Lcm6 (Table 1). In the next tailoring reaction aminosugar mycosamine is attached. Genes responsible for the biosynthesis of mycosamine and its attachment to the aglycone are present within all polyene BGCs, and the *lcm* gene cluster is no exception to this finding. Gene *lcm2* encodes a GDP-D-mannose 4,6-dehydratase, most probably responsible for the transformation of GDP-D-mannose into GDP-4-keto-6-deoxy-D-mannose. The latter undergoes spontaneous isomerization forming GDP-3-keto-6-deoxy-D-mannose (GDPDM). GDPDM is converted by aminotransferase Lcm5 into GDP-mycosamine. From the latter, the mycosamine residue is transferred onto polyene scaffold by glycosyltransferase Lcm4. Epoxidation is the last tailoring reaction, and Lcm10 is the most likely candidate for catalysis of this reaction^50^.

*Pathway-specific regulation and export.* All antifungal polyene BGCs described to date carry genes for transcriptional regulators^45^. The *pim* cluster, for instance, contains two such genes, PimR and PimM^51,52^, the nystatin BGC four: NysRI, NysRII, NysRIII and NysRIV^53^. PimR is a large multidomain protein known so far to have a single target within the *pim* cluster: *pimM.* PimM consists of a N-terminal effector PAS-like domain and a C-terminal LuxR-like DNA binding domain. PimM upregulates structural *pim* genes. In nystatin biosynthesis NysRI, NysRII and NysRIII are paralogs co-orthologous to PimR that play a redundant role in the activation of *nysRIV*, a *pimM* ortholog. The aforementioned pathway-specific regulators are crucial for the biosynthesis of respective compounds. Of note is that regulatory genes apparently are not within the sequenced part of the Lcm BGC of *S. achromogenes* NRRL 3125^47^. In our case the flank of the *lcm* gene cluster possesses three genes for the pathway-specific transcriptional regulators: *lcmRI, lcmRII* and *lcmRIII* (Table 1)*.* LcmRI is a NysRIV homolog, LcmRII and LcmRIII are homologs of PimM and PimR. There are also genes for ABC-transporters within the *lcm* cluster, *lcm8* and *lcm9*, encoding homologs of known ABC transporters involved in the export of pimaricin and amphotericin^54^. Finally, *lcm11* encodes a PimE homologue, which is functionally assigned to a cholesterol oxidase. The role of PimE remains obscure. It is crucial for pimaricin production and is believed to be a sensor which detects ergosterol in fungal membrane and somehow triggers the polyene export^55–57^.

### Relationships between AdpA, lucensomycin and landomycin biosynthetic pathways

To understand the mechanism of AdpA-mediated activation of Lcm biosynthesis, we analyzed the transcription of selected *lcm* genes in *S. cyanogenus* strains S136, S136 pGM4181^+^, ΔlanI7 and ΔlanI7 pGM4181^+^ by means of semi-quantitative RT-PCR. Chosen genes code for GDP-D-mannose 4,6-dehydratase (*lcm2*), monooxygenase (*lcm6*), ABC transporter (*lcm8*), fungal sensor (*lcm11*), key pathway-specific regulator (*lcmRIII*) and PKS (*lcmC*). Their products are involved in different steps of Lcm biosynthesis.

**Figure 4:**
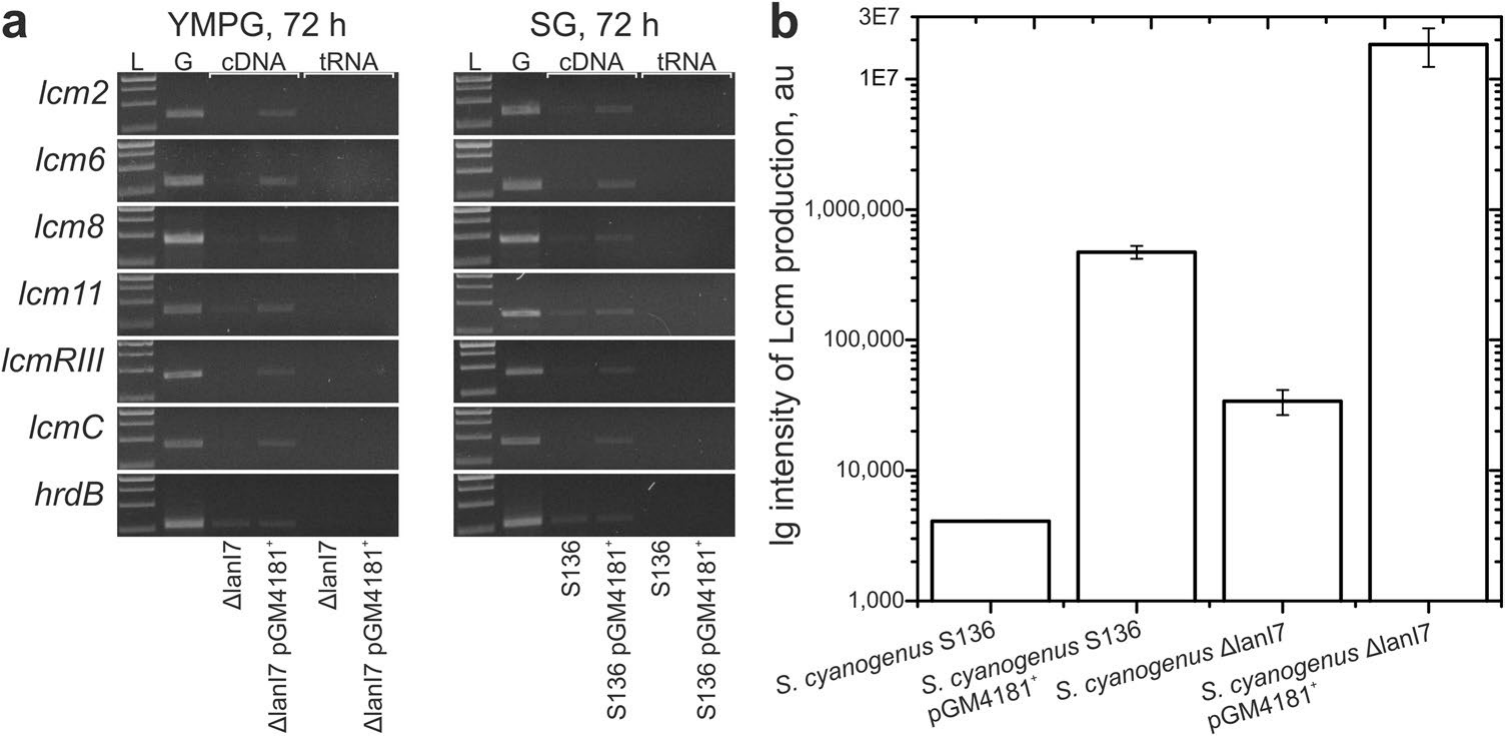
(**a**) Transcription of *lcm* genes involved in different stages of Lcm production (as well as house-keeping gene *hrdB*) in *S. cyanogenus* ΔlanI7 and ΔlanI7 pGM4181^+^ grown in YMPG medium for 72 h; *S. cyanogenus* S136 and S136 pGM4181^+^ grown in landomycin production SG medium. Genomic DNA isolated from S136 strain served as a positive control for the PCR-reactions (G). Total RNA samples from ΔlanI7, ΔlanI7 pGM4181^+^, S136 and S136 pGM4181^+^ were negative controls of the PCR reactions; (L) was 1 kbp DNA Ladder (Thermo Fisher Scientific, USA). (**b**) Quantification of Lcm production by *S. cyanogenus* S136, S136 pGM4181^+^, ΔlanI7 and ΔlanI7 pGM4181^+^ grown in YMPG for 120 h. Areas of Lcm mass peaks (708.35 Da (M + H)^+^) were integrated and represented as arbitrary units (au); please note logarithmic scale of the *y*-axis. Data represent mean values of three independent experiments ± 2SD.

First, we checked *lcm* gene expression in *S. cyanogenus* ΔlanI7 and *S. cyanogenus* ΔlanI7 pGM4181^+^ grown in YMPG for 72 h. The *lcm* transcripts were absent in *S. cyanogenus* ΔlanI7 and present in ΔlanI7 pGM4181^+^ strain (Fig. 4a). Next we analyzed the Lcm biosynthesis and *lcm* gene expression in landomycin-producing strains *S. cyanogenus* S136 and S136 pGM4181^+^ cultivated for 72 h in SG medium (optimal for landomycin A production) and YMPG (supports Lcm synthesis). In bioassays, strain S136 showed no signs of Lcm accumulation when grown on either SG or YMPG agar; weak antifungal activity was detected in case of YMPG-grown S136 pGM4181^+^ (ESM Fig. S7a). Although Lcm production by S136 pGM4181^+^ in SG was below detection levels (as evident from data in ESM Fig. S7b) the *lcm* genes were transcribed in the latter (Fig. 4a). We quantified the accumulation of Lcm by mass spectrometry in the extracts of different *S. cyanogenus* strains grown in liquid YMPG. The Lcm molecular mass signal was barely detectable in S136 and ΔlanI7. In S136 pGM4181^+^ and ΔlanI7 pGM4181^+^ Lcm increased roughly 100-fold (to (5.2 ± 0.5) × 10^5^ au) and 450-fold (to (1.9 ± 0.6 × 10^7^ au), respectively, as compared to their parental strains (Fig. 4b). Hence, in the wild type (S136) AdpA transcriptionally upregulates both Lcm (see above) and landomycin^36^ BGCs, yet only traces of Lcm can be detected. When the landomycin biosynthesis pathway is shut off (strain ΔlanI7), AdpA leads to abundant production of Lcm. Substantial quantitative differences in the levels of activation of Lcm production by S136 and ΔlanI7 most likely underlie qualitative differences in the bioassay results.

### Mechanism of AdpA-mediated regulation of lucensomycin production

The above findings show that AdpA is required for *lcm* gene expression and Lcm production in *S. cyanogenus.* To identify possible targets of AdpA*,* we analyzed the *lcm* cluster in silico for putative AdpA operator sites. Two adjacent high-scoring binding sites (TGGCGGAAAC, TGGCCGATAG; *p* < 10^−4^) were found within the *lcmRIII* promoter region. This, along with data on transcriptional activity of *lcm* genes (see above), allows to suggest that AdpA is likely to activate transcription of *lcm* genes by directly turning on *lcmRIII.* As in the case of pimaricin biosynthesis, LcmRIII should activate transcription of either *lcmRI* or *lcmRII*, or both. Since LcmRI and LcmRII are PimM/NysRIV-like regulators, at present it is difficult to deduce which one is responsible for the activation of *lcm* genes.

**Figure 5:**
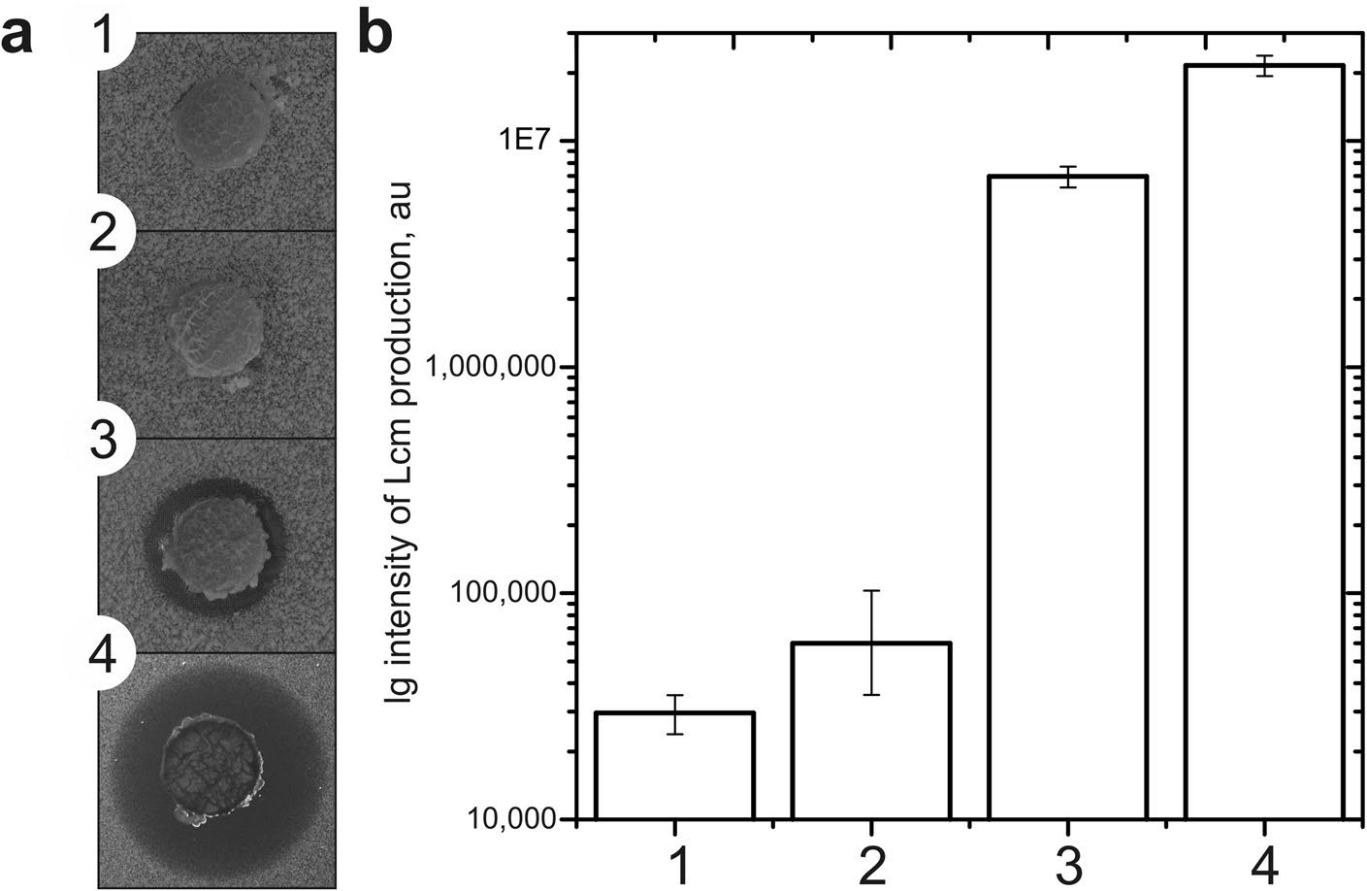
*D. hansenii* growth inhibition assay (**a**) revealed the activation of Lcm production by *S. cyanogenus* ΔlanI7 upon introduction of extra copy of *lcm* cluster-situated transcriptional regulators. Agar plugs were cut from the lawns of *S. cyanogenus* ΔlanI7 (1), ΔlanI7 *lcmRI*^+^ (2), ΔlanI7 *lcmRII*^+^ (3) and ΔlanI7 *lcmRIII*^+^ (4) grown on YMPG agar for 120 h. Lcm production by the same strains in liquid YMPG was quantified via LC–MS (**b**) in the three recombinant strains as compared to the initial one. Data represent mean values of three independent experiments, error bars are mean values ± 2SD.

To clarify the role of regulatory *lcm* genes, we overexpressed them individually under the control of *ermEp* in *S. cyanogenus* ΔlanI7 and determined Lcm production levels in the resulting strains. Lcm was observed in *S. cyanogenus* ΔlanI7 *lcmRIII*^+^ and *lcmRII*^+^*,* but not in *S. cyanogenus* ΔlanI7 *lcmRI*^+^ (Fig. 5a, b)*.* We therefore assume that AdpA has no targets in the *lcm* cluster other than *lcmRII* and *lcmRIII.* This assumption remains speculative and requires further investigations on AdpA binding to the *lcmRIII* promoter region. Gene *lcmRI* likely encodes a nonfunctional *lcmRII* paralog*.*

If low Lcm titers observed in the *adpA*-carrying S136 strain are caused by the concurrent production of landomycins, then a pathway-specific activation of the Lcm BGC should provide a competitive advantage for Lcm production, irrespective of the cultivation medium. Indeed, upon introduction of a *lcmRIII* expression construct into S136 we were able to detect significant antifungal activity of the latter when grown in media that support either landomycin (SG) or Lcm (YMPG) production, as well as on tryptic soy agar unfavorable for either biosynthetic route (Fig. S8a). At the same time, introduction of *lcmRIII* did not impact landomycin production by S136 (Fig. S8b).

### Variable termini of AdpA and a rare TTA codon are involved in high-level activation of Lcm biosynthesis

Results presented here and previously^35^ show that the isolated DNA binding domain of AdpA is capable of turning on gene expression. Yet, the contribution of different structural parts of AdpA to the efficiency of its function as an “activation tool” was not quantified. We have decided to build and test the allele of *adpA* lacking variable termini. Amino acid sequences of XNR_4181 (AdpA ortholog from *S. albus* J1074; 406 amino acids) and its 45 orthologs from the other *Streptomyces* were aligned, allowing for the identification of the most conserved region spanning from 15 to 350th aa residue of XNR_4181 (Fig. S9). We have cloned the sequence of *XNR_4181* coding the 15-350-aa stretch (XNR_4181i; Fig. S10) into pmoeE5script generating pGM4181i. Next, we cloned synthetic version of *XNR_4181* with TTA → CTC substitution^58^ into pmoeE5script generating pGM4181_tta_-. The recombinant *S. cyanogenus* ∆lanI7 strains carrying engineered *adpA* alleles produced Lcm on solid media as judged by the bioassays. Quantitative analysis of liquid cultures showed that removal of variable termini (pGM4181i) led to over tenfold decrease in Lcm production in comparison to ∆lanI7 pGM4181^+^. The Lcm production level of ∆lanI7 pGM4181_tta_-^+^ increased fivefold as compared to ∆lanI7 pGM4181i^+^, yet it still was two times lower than in ∆lanI7 pGM4181^+^ (Fig. S11).

## Discussion

**Figure 6:**
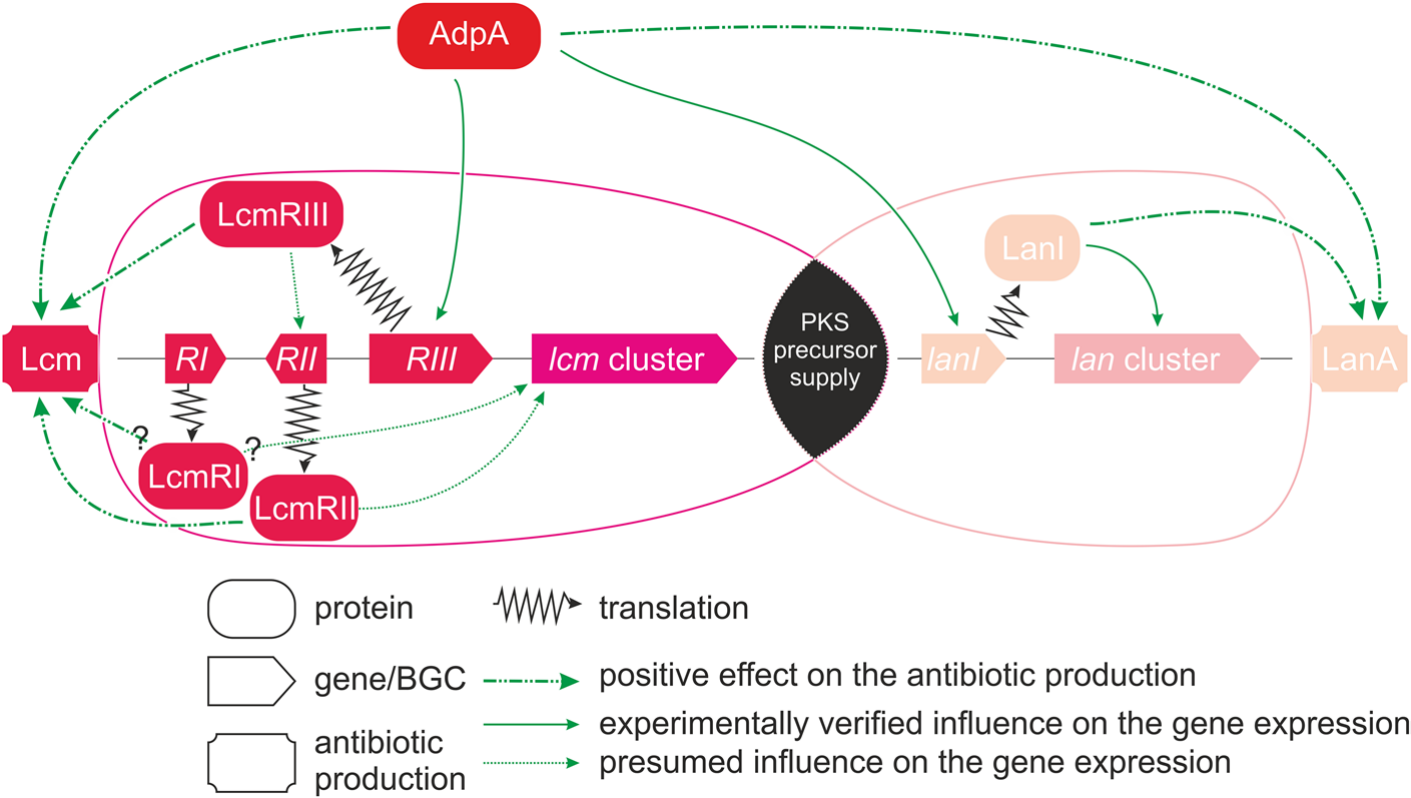
Proposal for the AdpA-mediated regulation of Lcm and the landomycin A biosynthetic pathways in *S. cyanogenus*. Both biosynthetic pathways compete for PKS precursor supply. AdpA, presumably through direct binding to the *lcmRIII* promoter region, activates the expression of this *lcm* cluster-situated transcriptional regulator. Both LcmRII and LcmRIII are positive regulators of Lcm production. The role of LcmRI in the regulation of the Lcm biosynthesis is unclear and awaits further investigations.

Over the last two decades of investigations it has become clear that the vast majority of secondary metabolite BGCs within actinobacterial genomes is silent^59,60^. As summarized in the introductory part, the obstructed transcription appears to be the key reason for the BGCs to be silent, and researchers proposed numerous approaches to overcome this obstacle. There is a recent report on the activation of silent BGCs in actinomycetes via knockouts of genes for active secondary metabolic pathways, implying that precocious production of one metabolite diminishes the chances for the other to be produced^61^. Our study, summarized in Fig. 6, describes a more complex case of successful activation of a silent pathway requiring its transcriptional activation, elimination of the competing active pathway, and appropriate cultivation medium. A few aspects of our findings merit further comments.

Our previous results^36^ showed that overexpression of heterologous *adpA* genes in *S. cyanogenus* S136 enhances the transcription of *lan* genes and improves landomycin A production. The above mentioned study and the results reported here support the idea that native AdpA of *S. cyanogenus* is non-functional. The landomycin A biosynthetic route benefits from functional AdpA, but is not dependent on it. In contrast, the Lcm biosynthetic pathway remains silent in the absence of functional *adpA*. RT-PCR analysis shows that in the wild type *S. cyanogenus* S136 (as well as in *S. cyanogenus ∆*lanI7) the transcription of *lcm* genes is enhanced upon introduction of functional *adpA*. Yet in case of *S. cyanogenus* S136 (and unlike in ∆lanI7) this did not cause Lcm production high enough to suppress fungal growth in the bioassays. This phenomenon might be explained by the competition between landomycin and Lcm biosynthetic pathways for the common pool of acyl-CoA units. If the landomycin biosynthetic route is silenced (as it is the case for mutant ∆lanI7), the precursor pool becomes available for the Lcm pathway. Such scenario, however, does not explain the absence of Lcm production in *S. cyanogenus* S136 pGM4181^+^, where *lcm* genes are expressed at the same level as in *∆*lanI7 pGM4181^+^ and Lcm is actively produced. The reasons for and mechanisms of such “preferences” for the landomycin biosynthetic route in *S. cyanogenus* at the expense of the other secondary metabolic pathways remain obscure and require further investigations. These findings demonstrate that transcription of genes for secondary metabolism might be inconsequential to the production of secondary metabolites themselves. Altogether our results caution against overt reliance on transcriptome and translatome data as a proxy to *phenotypic* expression of actinobacterial BGCs^13,62,63^.

AdpA-activated expression of *lcm* BGC is mediated with the activation of *lcmRIII,* coding for a *lcm* cluster-situated transcriptional regulator. Transcription of this gene appears to be strictly dependent on *adpA* and putative AdpA binding sites were found within its promoter region. We note that AdpA was also shown to be involved in regulation of two other polyene antibiotics, pimaricin and candicidin^64,65^. Nevertheless, the location of AdpA binding sites relative to coding sequences cannot serve as a predictor of its involvement into or a sign of effects on secondary metabolic pathways^66,67^. Thus, more experiments are required to unambiguously prove that *lcmRIII* is a major and direct target of AdpA in context of Lcm biosynthesis. These experiments are underway in our laboratories.

Besides the importance of a DNA-binding domain for AdpA function, little is known about other structural features of AdpA that influence its regulatory capabilities. Here we show that removal of N- and C-terminal variable regions of AdpA affected its ability to upregulate Lcm production. One possibility is that AdpA with shortened termini is not able to form dimers. This inability would not block regulatory properties of AdpA (as the expression of separate DNA-binding domain of AdpA showed), but may lower the affinity of its binding to operator sequences. Also, the shortened AdpA might not be able to efficiently recruit RNA polymerase to form the open complex at the promoter regions. The *adpA* allele featuring synonymous TTA → CTC substitution was also less efficient in the activation of Lcm biosynthesis. The TTA codon is thought to be a part of a regulatory mechanism that limits the level of AdpA protein at the translational level^68^. For a number of TTA-containing regulatory genes of *Streptomyces* the substitution of this codon for a more abundant synonym caused earlier and more abundant production of respective regulatory protein^69^. Nevertheless, the role of the TTA codon in *adpA* remains elusive. AdpA is not produced in mutants devoid of tRNA for the UUA codon^70–72^, yet strains expressing TTA^+^ and TTG^+^ *adpA* alleles did not differ in temporal patterns of AdpA accumulation^34^. The rather detrimental effect of TTA → CTC substitution on Lcm production suggests that TTA could be more than a switch to control the onset/level of AdpA production. For example, rare codons within interdomain linkers of bacterial mRNAs could slow down protein synthesis of the ribosome and thus optimize co-translational protein folding^73^.

Activation of Lcm BGC in *S. cyanogenus* led to the first description of the entire biosynthetic machinery behind the assembly of this natural product. It is similar to other antifungal polyene (e.g. pimaricin, nystatin, rimocidin etc.) BGCs in many respects, making it straightforward to ascribe the function to most *lcm* genes. The organization of initiation module LcmA protein is a distinguishing feature of Lcm PKS. We tentatively suggest that this module is responsible for the generation of the side propyl chain of the lucensomycin aglycone. Further investigations are in progress to verify the proposed mechanism and to understand as to whether LcmA can be used to modify the other polyene antibiotics.

The identity of the other novel mass peaks observed in *adpA*-expressing ∆lanI7 strain remains unknown. This emphasizes one another formidable challenge in the area of natural product research: signals of novel chemical compounds detected in biological samples are often not pursued if there are no ways for their robust production in reasonable quantities for downstream analysis. Our report stands in a long line of the other “success stories” where a single product was activated in a particular strain (in response to a certain procedure), leaving out of focus the factors that limit the expression of the rest BGCs in that strain. Nevertheless, our work provides a rationale for a new research venue to solve this issue. Particularly, much like the elimination of expression of the landomycin BGC helped us to produce and characterize Lcm, the engineered silencing of Lcm BGC may lead to increased production of the other as-yet-unknown metabolites. We believe that a more systematic investigation of the effects of active and activated BGCs on the silent metabolome of single *Streptomyces* strain is worth the effort. This will yield new insights into complex interactions between the pathways at genetic and metabolic levels, eventually leading to a fuller exploitation of chemical diversity hidden in bacterial genomes.

## Methods

### Bacterial strains, plasmids and cultivation conditions

Bacterial strains and plasmids, used in this work, are described in ESM Table S1. For intergeneric conjugations, *S. cyanogenus* strains were grown on ISP3 or SFM agar^74^ at 30 °C. Characterization of antimicrobial activities of recombinant *S. cyanogenus* ΔlanI7 derivatives was done on different solid media described in ESM. The same amounts of *S. cyanogenus* mycelia ((0.8 ÷ 1) × 10^7^ c.f.u.) were plated to produce lawns for the bioassays. For submerged production of Lcm, *S. cyanogenus* strains were grown in YMPG medium (please see ESM). Where required, the strains were maintained in presence of apramycin sulphate (50 μg/mL).

### Bioassays

Agar plug antibiotic diffusion assay was used to determine antimicrobial activities of *S. cyanogenus* strains grown on agar media listed in ESM. In all cases plates contained 30 mL of solid medium. To assay antifungal activity, agar plugs (Ø 5 mm) were cut from *S. cyanogenus* lawns and placed on the surface of TSB agar plates with freshly seeded 10^6^ cells of *D. hansenii*. The antibacterial activity was similarly tested; here plugs were stacked onto modified minimal medium (g/L: KH_2_PO_4_—3, K_2_HPO_4_—7, sodium citrate × 4H_2_O—0.5, MgSO_4_ × 7H_2_O—0.1, (NH_4_)_2_SO_4_—1, glucose—2, bacto peptone—0.3, agar—16;^75^) with *B. cereus* ATCC 19637 spores (10^7^ per plate); or LB agar with *E. coli* DH5α cells (10^7^ per plate). Disc diffusion assay was also employed. Here 5 mm Whatman paper discs were soaked into methanol extracts containing lucensomycin and placed on the surface of TSB agar plates with 10^6^ cells of *D. hansenii.* Unless otherwise stated, in all cases the extracts were obtained from the same amount of biomass (400 mg, wet weight). Bioassay plates were incubated at 37 °C (30 °C in the case of *D. hansenii*), halos of growth inhibition around the plugs or discs were analyzed after 20 h of incubation.

### Submerged production, purification and LC–MS of Lcm

250-mL flasks containing 50 mL of TSB were inoculated with agar slices cut from 168 h old *S. cyanogenus* lawns grown on ISP3 agar. The flasks were incubated for 72 h on the rotary shaker at 30 °C, 200 rpm. 1.5 mL of the resulting culture was transferred into 250-mL flasks containing 50 mL of YMPG medium. Main cultures were grown for up to 120 h at 30 °C, 200 rpm. For analytical purposes, 1 g of wet biomass was washed three times with deionized water and extracted with 2 mL of methanol. To determine optimal fermentation time for Lcm production, biomass was collected from the main culture at five time points. Extracts were concentrated to the volume of 100 μL and 40 μL of were subjected to paper disc diffusion assay (see above)*.*

Scaled-up purification of Lcm was done as follows. Four 2-L flasks with steel springs containing 500 mL of YMPG were inoculated with 8 mL of preculture (*S. cyanogenus* ∆lanI7 pGM418^+^; prepared as described above) and grown for 72 h on the rotary shaker at 30 °C, 200 rpm. Biomass and spent medium were separated with centrifugation. Cells from 2 L of the cultures were washed with water and resuspended in 160 mL of methanol:acetone (1:1). The resulting mixture was sonicated for 15 min, spun down; supernatant was filtered through the 3 mm paper. The spent medium was mixed with ethyl acetate (1:1) by inversion a few times, then the mixture was left to allow for the phases to separate. The organic extracts from cells and supernatant were combined and evaporated *in vacuo*. Dry residue was dissolved in methanol and separated on preparative HPLC column (conditions were the same as for analytical LC–MS, see below). Fractions having an absorption maximum at λ = 310 nm (characteristic for polyene compounds) eluted at 7.4- ÷ 8.4 min. The presence of the 708.35 Da (Lcm) mass peak in this fraction was verified with LC–MS. The collected fraction was evaporated to dryness, giving approximately 100 mg of pale pink solid. Upon addition of 4 mL methanol a white precipitate was formed, which was shown via LC–MS to consist of pure 708.35 Da compound. As a result, we were able to purify 40 mg of Lcm from 2-L flask fermentation. Whenever possible, all plastic- and glassware was wrapped in aluminum foil to eliminate light-induced degradation of Lcm.

Methanol-dissolved Lcm samples were analyzed with HPLC–MS on a HiRes Extractive Oribtrap MassSpectrometer (Thermo Fisher Scientific). The solvent system was water + 0.1% HFo (solvent A); acetonitrile + 0.1% HFo (solvent B); 95% A to 0% A in 8 min, then 1.5 at 0% A, then back to 95% A in 1.5 min. Data analysis was carried out using Xcalibur 5.1 software (Thermo Fisher Scientific).

### Plasmid construction

Fragment of the *XNR_4181* coding for DNA-binding domain (700–1245 bp) was amplified using xnr4181araC_up and xnr4181_EcoRIrp primers and cloned into SpeI/EcoRI sites of pmoeE5script^76^ yielding pGM4181d. Gene fragments coding for DNA-binding domains of *adpAsco*, *adpAscla* and *adpAgh* were cloned similarly using primers described in Table 2, generating pGMSCOd, pGMSCLAd, pGMSGHd. To construct pGM4181i, fragment of *XNR_4181* coding sequences from 46 to 1019 bp was amplified using iAdpA_F/R primer pair from pGM4181 and cloned into SpeI/EcoRI sites of pmoeE5script. pGM4181_tta-_ was constructed in a similar fashion, using xnr4181_XbaIup/xnr4181_EcoRIrp primer pair to amplify *XNR_4181*_*tta-*_ coding sequence from pUCXNR. Pathway-specific regulatory genes *lcmRI, lcmRII* and *lcmRIII* were amplified from the chromosome DNA of *S. cyanogenus* S136 using primers listed in Table 2 and cloned into XbaI/EcoRI sites of pTES^77^, yielding pTES22, pTES23 and pTES25 respectively.

**Table 2:** Primers used in this work.

Primer	Nucleotide sequence (5′–3′), recognition sites for restriction endonucleases are underlined in primer sequence	Purpose
iAdpA_F	AAATCTAGACGAGCAACGGAGGTACGGACATGAAACTCTCCGGGCGGCGCC	*XNR_4181i* cloning
iAdpA_R	AAAGAATTCTCACGGACGGCGGGCCCGGTAGG	-//-
xnr4181_XbaIup	AAATCTAGAGGGGGGCTTAGTCACATG	*XNR_4181*_*tta*_- cloning
xnr4181_EcoRIrp	AAAGAATTCGGAGCTGTCCTCTCTCAGAC	-//-
Xnr4181araC_up	AAATCTAGACGAGCAACGGAGGTACGGACATGCCGGAGGAGATCGGCGCCGAC	*adpA*_*scy*_*dbd/ adpA*_*sgh*_*dbd/ adpA*_*sco*_*dbd* cloning
SCLAd_F	AAATCTAGACGAGCAACGGAGGTACGGACATGCCAGAGGAAATCGGGTCGGAC	*adpA*_*scla*_*dbd* cloning
SCLAVADPA_R	AAAGAATTCCATGCGACTACCTTATGG	*adpA*_*scla*_*dbd* cloning
SCOADPA_R	AAAGAATTCGCCGTCTGCTCACCTCACGG	*adpA*_*sco*_*dbd* cloning
SGHADPA_R	AAAGATATCGCCTCCGGCCCCGTCCGGTGT	*adpA*_*sgh*_*dbd* cloning
gbc22_F	TTTTCTAGAGGAGGGTCGGAATGGCGGCGCTGG	*lcmRI* cloning
gbc22_R	TTTGAATTCGGGGTTTCAGCCGGACTCG	-//-
gbc23_F	TTTTCTAGAGGAGGCACACCGTGATTGACGAAAC	*lcmRII* cloning
gbc23_R	TTTGAATTCTCCGCTCCCTGTCGTCCTC	-//-
gbc25_F	TTTTCTAGAGGAGGACCGTCATGCCCGTACCCC	*lcmRIII* cloning
gbc25_R	TTTGAATTCGACCGCGCGAGCGGTGGCTC	-//-
amR_F	CGGGGTCTGACGCTCAGTGGA	*aac(3)IV* amplification
amR_R	AGCGTCTGCTCCGCCATTCG	-//-
rt_hrdB_fp	GCTGGCCAAGGAGCTCGAC	278 bp fragment of *hrdB* (RT-PCR)
rt_hrdB_rp	CGTCGAGGGTCTTCGGCTG	-//-
L2_F	ACGCCGGTCCCGATCACGTA	355 bp fragment of *lcm2* (RT-PCR)
L2_R	TCGGGAAGGTCGCGGAGACT	-//-
L6_F	CGAGTCGCGCACCAAAACGC	312 bp fragment of *lcm6* (RT-PCR)
L6_R	TAGCGCAGCAGCTCGTCCAC	-//-
L8_F	GGTCGGCCGCGATCTGAGGT	446 bp fragment of *lcm8* (RT-PCR)
L8_R	CCCTGCGGGTCAGCACGAAC	-//-
L11_F	CTGCGCTGCCGGATCGTCTT	380 bp fragment of *lcm11* (RT-PCR)
L11_R	TTCGAGGAGGTGCTGCCCCA	-//-
LRIII_F	CGTCACGGATCGCGGGCAC	430 bp fragment of *lcmRIII* (RT-PCR)
LRIII_R	CGTGCTTTCCAGACGTGCGG	-//-
lPKS_F	TGTTGCCCGAGAGCCTCACG	420 bp fragment of *lcmC* (RT-PCR)
lPKS_R	CTACGGCCGCAACCGCCCC	-//-

Recognition sites of the restriction endonucleases used for the cloning are underlined.

### Generation of recombinant *Streptomyces* strains

All constructs were transferred into *S. cyanogenus* ΔlanI7 by intergeneric conjugation with *E. coli* ET12567 pUZ8002^+^, as described elsewhere^74^. *S. cyanogenus* and transconjugants carrying pmoeE5script- and pTES-based plasmids were selected on plates overlaid with 50 μg/mL of apramycin sulphate and 30 μg/mL of nalidixic acid. Apramycin-resistant transconjugants were verified with PCR and primers complementary to marker *aac(3)IV* gene (Table 2).

### Total RNA isolation and semi-quantitative RT-PCR analysis

Samples of *S. cyanogenus* mycelium were collected from 5 mL of culture broth by centrifugation (6000 rpm, 5 min, 4 °C). RNA isolation and cDNA synthesis was performed as described previously^36^. PCR was performed using Phusion High-Fidelity DNA polymerase (Thermo Fisher Scientific) and primer pairs specific to each individual gene (Table 2). As a positive control for gene expression, the primer pair specific to *S. cyanogenus* house-keeping gene *hrdB* was used. PCR products were analyzed by electrophoresis in 2% TAE agarose gel.

### DNA sequencing and in silico analysis

Initially, *S. cyanogenus* genome was sequenced using Illumina approach as described in^36^. However, the initial sequence of *lcm* cluster contained large gaps in the one of the *lcm* PKS genes, known to be challenging target to sequence due to their highly repetitive nature. Therefore, in this work *S. cyanogenus* S136 genome was re-sequenced using a combination of HiSeq Illumina and GridION ONT technologies, essentially as described in^78^. Final S136 genome (8773 kbp) was manually curated and annotated using Prokka annotation pipeline^79^. Full sequence of S136 genome will be published separately.

Annotation of putative BGCs on the chromosome of *S. cyanogenus* was achieved with antiSMASH^14^. Comparison of the *lcm* cluster with already described polyene antibiotic BGCs was made with the assistance of DoBISCUIT database^80^. PKS modules were predicted using PKS/NRPS Analysis website^81^. Search for conserved motifs within the *lcm* cluster was carried out with the help of the MEME suite^82^.

## Supplementary Information

Supplementary Information.
