# Supplementary material for: Eliciting the silent lucensomycin biosynthetic pathway in Streptomyces cyanogenus S136 via manipulation of the global regulatory gene adpA
Source: Sci Rep. 2021 Feb 10;11:3507. doi: 10.1038/s41598-021-82934-6 (PMC7875965; doi:10.1038/s41598-021-82934-6)

Scientific Reports

Eliciting the silent lucensomycin biosynthetic pathway in *Streptomyces cyanogenus* S136 via manipulation of the global regulatory gene *adpA*

Oleksandr Yushchuk[1], Iryna Ostash[1], Eva Mösker[2], Iryna Vlasiuk[1], Maksym Deneka[1], Christian Rückert[3], Tobias Busche[3], Victor Fedorenko[1], Jörn Kalinowski[3], Roderich Süßmuth[2*] and Bohdan Ostash[1*]

[1]Department of Genetics and Biotechnology of Ivan Franko National University of Lviv, 4 Hrushevskoho st., Lviv 79005, Ukraine

[2]Institut für Chemie, Technische Universität Berlin, Straße des 17. Juni 124, 10623 Berlin, Germany

[3]Technology Platform Genomics, CeBiTec, Bielefeld University, Universitätsstraße 27, 33615 Bielefeld, Germany

***Corresponding authors:**
For biology: Prof. B. Ostash
Ivan Franko National University of Lviv
Department of Genetics and Biotechnology,
Hrushevskoho st. 4, Rm. 102,
Lviv 79005
Tel.: +38-036-3973036
e-mail: bohdan.ostash@lnu.edu.ua

For chemistry: Prof. Dr. R. Süßmuth
Technische Universität Berlin,
Institut für Chemie,
Straße des 17. Juni 124
Berlin 10623Tel.: +49-030-31424205
e-mail: suessmuth@chem.tu-berlin.de

Electronic Supplementary Materials

Inventory of Supplementary Files

Supplementary Materials and Methods
1. Media recipes

Supplementary Figures and Tables
Table S1. Strains and plasmids used in the work
Fig. S1. Natural product BGCs (similar to known ones) found within the chromosome of *S. cyanogenus*.
Fig. S2. AdpA-induced expression of melanin biosynthesis genes in *S. cyanogenus.*
Fig. S3. AdpA-induced antifungal activity in *S. cyanogenus* grown on different solid media.
Fig. S4. Secondary metabolomes of *S. cyanogenus* S136 and S136 pGM4181$^+$ in YMPG.
Table S2. Analysis of antibiotic activity of the pure 708.35 Da compound against bacteria and fungi.
Fig. S5. Activity of *S. cyanogenus* ΔlanI7 pGM4181$^+$ against *Fusarium oxisporum.*
Fig. S6. MS-MS analysis of 708.35 Da compound produced by *S. cyanogenus* ΔlanI7 pGM4181$^+$.
Fig. S7. Comparison of AdpA-induced Lcm production by *S. cyanogenus* S136 pGM4181$^+$ and ΔlanI7 pGM4181$^+$.
Fig. S8. Activation of Lcm production by *S. cyanogenus* S136 via overexpression of *lcmRIII* pathway-specific regulator.
Fig. S9. Multiple amino acid sequence alignment of different AdpA alleles.
Fig. S10. Multiple nucleotide sequence alignment of $adpA_{sco}$, *XNR_4181* and the new alleles of the latter.
Fig. S11. Novel *adpA* alleles activate Lcm production by *S. cyanogenus* ΔlanI7.

Supplementary References

Non-cropped versions of agarose gels from main text Fig. 4

Original version of the agarose gel present in ESM, Fig. S2

Supplementary Materials and Methods

Media used in this work

MS, SMMS - Kieser et al. 2000

ISP2 (g/L):
yeast extract – 4;
malt extract (Difco) – 10;
dextrose – 4;
agar – 20;
pH 7.2.

ISP3 (g/L):
finely ground whole oats (tolokno; Kozub®, Ukraine) – 30;
agar – 18;
tapped water – to 1L, pH 8.0 prior to autoclaving (with NaOH).

ISP4 (g/L):
Soluble starch – 10;
$CaCO_3$ – 2;
K_2HPO_4 (anhydrous) – 1;
$MgSO_4$ x 7 H_2O – 1;
NaCl – 1;
$(NH_4)_2SO_4$ – 2;
$FeSO_4 \times 7\ H_2O$ – 0.001;
$MnCl_2 \times 4\ H_2O$ – 0.001;
$ZnSO_4 \times 7\ H_2O$ – 0.001;
Agar – 20;
pH 7.0 – 7.4.

ISP5 (g/L):
L-Asparagine – 1;
K_2HPO_4 – 1;
glycerol – 10;
$FeSO_4 \times 7H_2O$ – 0.001;
$MnCl_2 \times 4H_2O$ – 0.001;
$ZnSO_4 \times 7H_2O$ – 0.001;
Agar – 20;
pH 7.0.

TSB from Himedia (g/L):
pancreatic digest of casein – 17;
papaic digest of soybean meal – 3;
NaCl – 5;
K_2HPO_4 – 2.5;
glucose – 2.5.

SG (g/L):
glucose – 20;
soytone (Difco) – 10;
$CaCO_3$ – 2;
$CoCl_2$ – 0.001;
pH 7.2.

YMPG (g/L):
yeast extract – 4;
bacto peptone – 1;
malt extract – 10;
glucose – 10;
$MgCl_2 \times 6H_2O$ – 2;
pH 7.0.
Add 20 g of agar to obtain solid YMPG.

Bennet's agar (g/L):
glucose – 10;
bacto peptone – 1;
yeast extract – 1;
tryptone – 2;
agar – 20;
pH 7.2.

MYM (g/L):
yeast extract – 4;
malt extract – 10;
maltose – 4;
agar – 20;
pH 7.2.

Table S1. Strains and plasmids used in the work

Name	Description (Am^r, Sp^r, Ap^r are marker genes for apramycin, spectinomycin and ampicillin resistance respectively)	Source or reference
E. coli DH5α	General cloning host	MBI Fermentas
E. coli ET12567 (pUZ8002)	(*dam-13*::Tn9 *dcm-6*), pUZ8002$^+$ (Δ*oriT*), used for conjugative transfer of DNA	Kieser et al., 2000
D. hansenii VKM Y-9	*Saccharomycetales* yeasts, test culture	VKM
B. cereus ATCC19637	Gram-positive bacterium, test culture	ATCC
S. cyanogenus S136	Wild type, producer of landomycin A	DSMZ
S. cyanogenus pGM4181$^+$	*S. cyanogenus* S136 derivative carrying pGM4181	Yushchuk et al., 2018
S. cyanogenus ΔlanI7	*S. cyanogenus* S136 with the knockout of pathway-specific regulatory gene *lanI*	Rebets et al., 2008
S. cyanogenus ΔlanI7 pGM4181$^+$	*S. cyanogenus* ΔlanI7 derivative carrying pGM4181	Yushchuk et al., 2018
S. cyanogenus ΔlanI7 pGM4181d$^+$	*S. cyanogenus* ΔlanI7 derivative carrying pGM4181d	This work
S. cyanogenus ΔlanI7 pGM4181i$^+$	*S. cyanogenus* ΔlanI7 derivative carrying pGM4181i	This work
S. cyanogenus ΔlanI7 pGM4181$_{tta}$-$^+$	*S. cyanogenus* ΔlanI7 derivative carrying pGM4181$_{tta}$-	This work
S. cyanogenus ΔlanI7 pGMSCY	*S. cyanogenus* ΔlanI7 derivative carrying pGMSCY	This work
S. cyanogenus ΔlanI7 pGMSCYd	*S. cyanogenus* ΔlanI7 derivative carrying pGMSCYd	This work
S. cyanogenus ΔlanI7 pGMSCLA	*S. cyanogenus* ΔlanI7 derivative carrying pGMSCLA	This work
S. cyanogenus ΔlanI7 pGMSCLAd	*S. cyanogenus* ΔlanI7 derivative carrying pGMSCLAd	This work
S. cyanogenus ΔlanI7 pGMSCO	*S. cyanogenus* ΔlanI7 derivative carrying pGMSCO	This work
S. cyanogenus ΔlanI7 pGMSCOd	*S. cyanogenus* ΔlanI7 derivative carrying pGMSCOd	This work
S. cyanogenus ΔlanI7 pOOB95d	*S. cyanogenus* ΔlanI7 derivative carrying pOOB95d	This work
S. cyanogenus ΔlanI7 pGMSGHd	*S. cyanogenus* ΔlanI7 derivative carrying pGMSGHd	This work
S. cyanogenus ΔlanI7 *lcmRI*$^+$	*S. cyanogenus* ΔlanI7 derivative carrying pTES22	This work
S. cyanogenus ΔlanI7 *lcmRII*$^+$	*S. cyanogenus* ΔlanI7 derivative carrying pTES23	This work
S. cyanogenus ΔlanI7 *lcmRIII*$^+$	*S. cyanogenus* ΔlanI7 derivative carrying pTES25	This work
pTES	ϕC31-based integrative expression vector containing *ermEp* (Am^r)	Herrman et al., 2012
pmoeE5script	pGUS derivative containing moeE5p cloned upstream of *gusA* (Am^r Sp^r)*	Makitrynskyy et al. 2013
pGM4181	pmoeE5script with *XNR_4181* cloned instead of *gusA*	Yushchuk et al. 2018
pGM4181d	pmoeE5script with *XNR_4181dbd* cloned instead of *gusA*	This work
pGMSCLA	pmoeE5script with $adpA_{scl}$ cloned instead of *gusA*	Yushchuk et al. 2018
pGMSCLAd	pmoeE5script with $adpA_{scl}dbd$ cloned instead of *gusA*	This work
pGMSCO	pmoeE5script with $adpA_{sco}$ cloned instead of *gusA*	Yushchuk et al. 2018
pGMSCOd	pmoeE5script with $adpA_{sco}dbd$ cloned instead of *gusA*	This work
pOOB95d	pmoeE5script with $adpA_{ah}$ cloned instead of *gusA*	Yushchuk et al. 2018
pGMSGHd	pmoeE5script with $adpA_{ah}dbd$ cloned instead of *gusA*	This work
pGMSCY	pmoeE5script with $adpA_{scy}$ cloned instead of *gusA*	Yushchuk et al. 2018
pGMSCYd	pmoeE5script with $adpA_{scy}dbd$ cloned instead of *gusA*	Yushchuk et al. 2018
pTES22	pTES derivative carrying *lcmRI* under the control of *ermEp*	This work
pTES23	pTES derivative carrying *lcmRII* under the control of *ermEp*	This work
pTES25	pTES derivative carrying *lcmRIII* under the control of *ermEp*	This work
pUCXNR	pUC19 carrying the synthetic XNR_4181_{tta}-, Ap^r	Koshla et al. 2019
pGM4181i	pmoeE5script with *XNR_4181i* cloned instead of *gusA*	This work
pGM4181$_{tta}$-	pmoeE5script with XNR_4181_{tta}- cloned instead of *gusA*	This work

Supplementary Figures and Tables

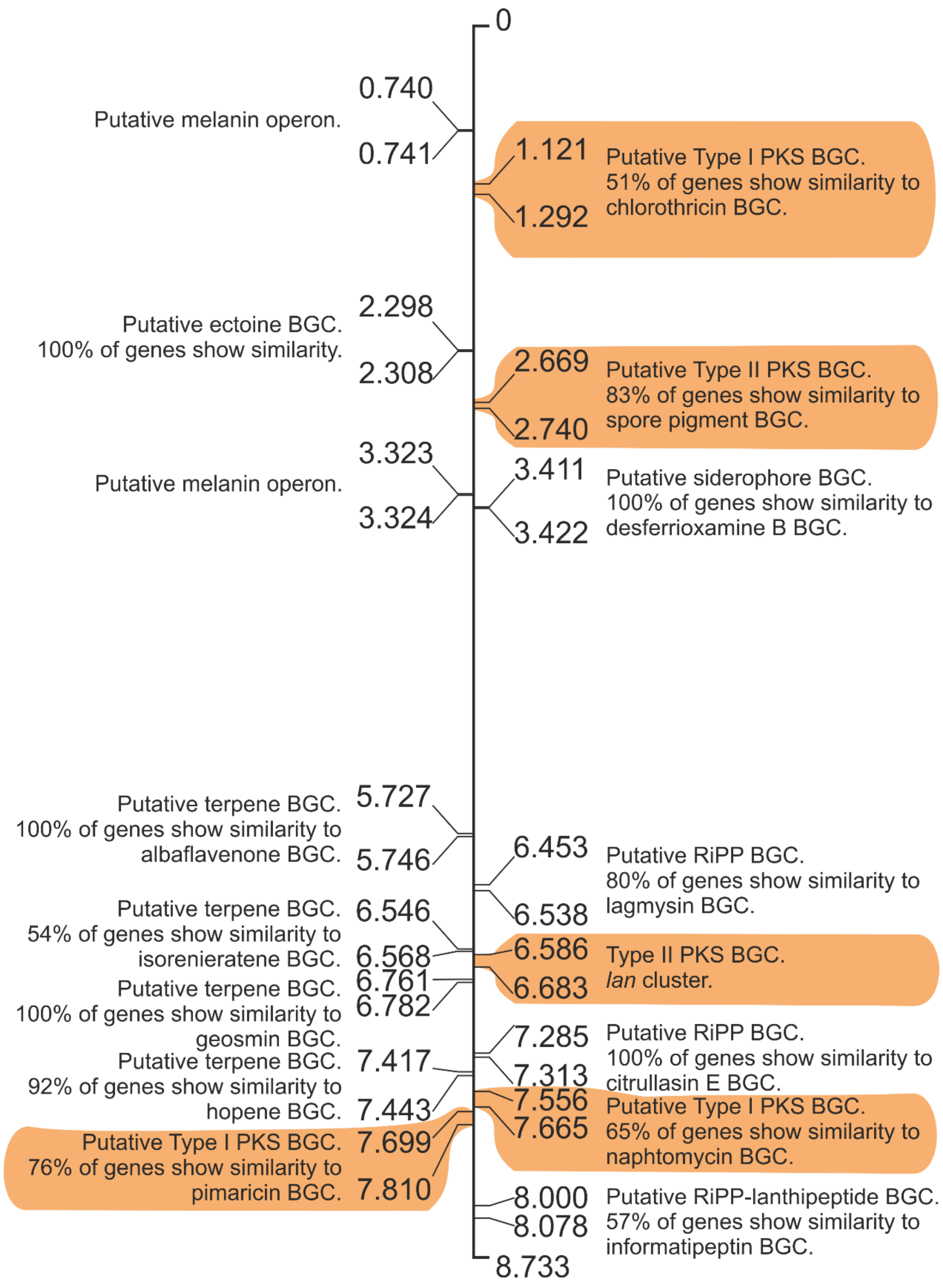

Fig. S1. Schematic representation of secondary metabolite biosynthetic gene clusters (BGCs) revealed on the chromosome of *S. cyanogenus* S136 using antiSMASH. The polyketide BGCs are highlighted (light-brown background).

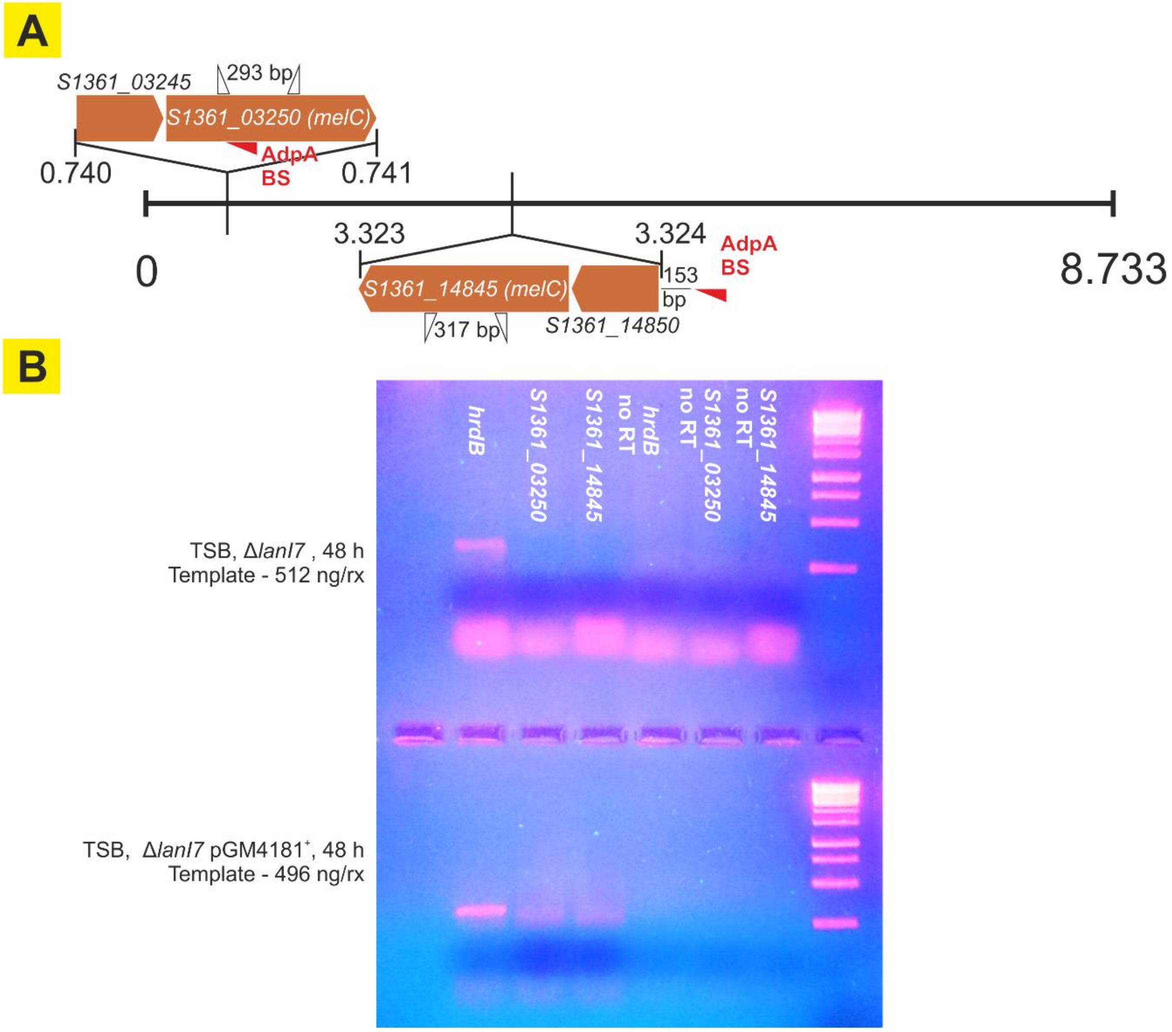

Fig. S2. Allocation of the genes, putatively involved in the biosynthesis of melanoid pigments, on the chromosome of *S. cyanogenus* (**A**). Red triangles label the location and orientation of putative AdpA binding sites (for S1361_03250: TGGCCGGTC, for S1361_14845: TGGCCCGAT). Expression of *S1361_03250* and *S1361_14845* genes is activated in *XNR_4181 (adpA* gene from *S. albus)* overexpressing strain (ΔlanI7 pGM4181^{+}, **B**), as measured by semi-quantitative RT-PCR. Fragments amplified during RT-PCR are marked with open triangles. The photo was uniformly contrasted (PowerPoint drawing settings) to emphasize the bands in pGM4181-expressing strain; original photo is shown on the last page of this ESM file. Positive control is *hrdB* gene for major vegetative sigma factor in *Streptomyces* (e.g. *SCO5820* in *S. coelicolor* M145)

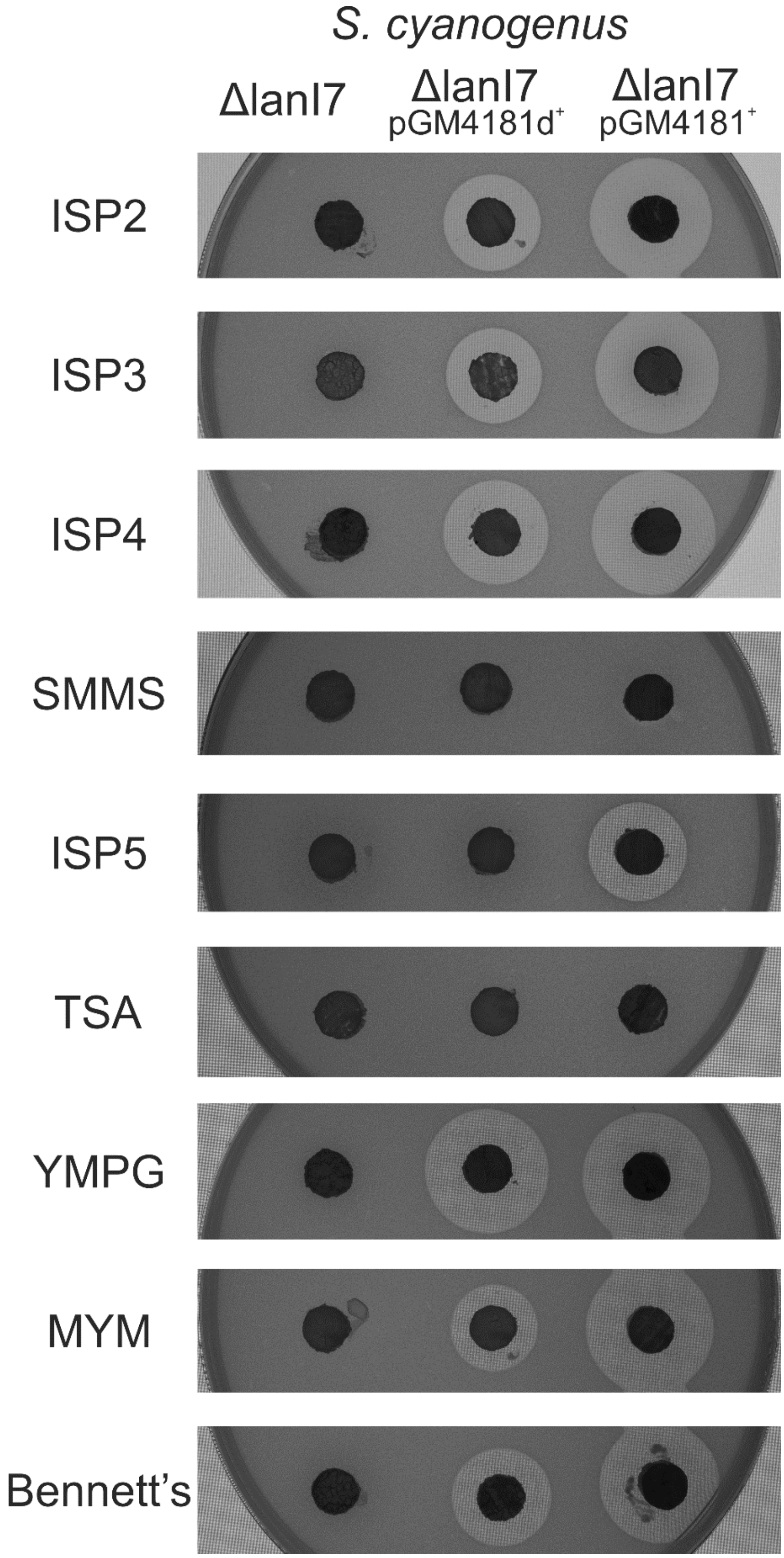

Fig. S3. Induction of antifungal activity in S. *cyanogenus* upon introduction of *XNR_4181* (pGM4181) and *XNR_4181dbd* (pGM4181d) is medium-dependent. *D. hansenii* was used as a fungal culture in agar plug assay, as described in Methods of the main text. Conditions of *S. cyanogenus* cultivation: 120 h of growth, 30 °C, type of the medium is noted to the left of the photos. Photos represent typical result of four biological replicates.

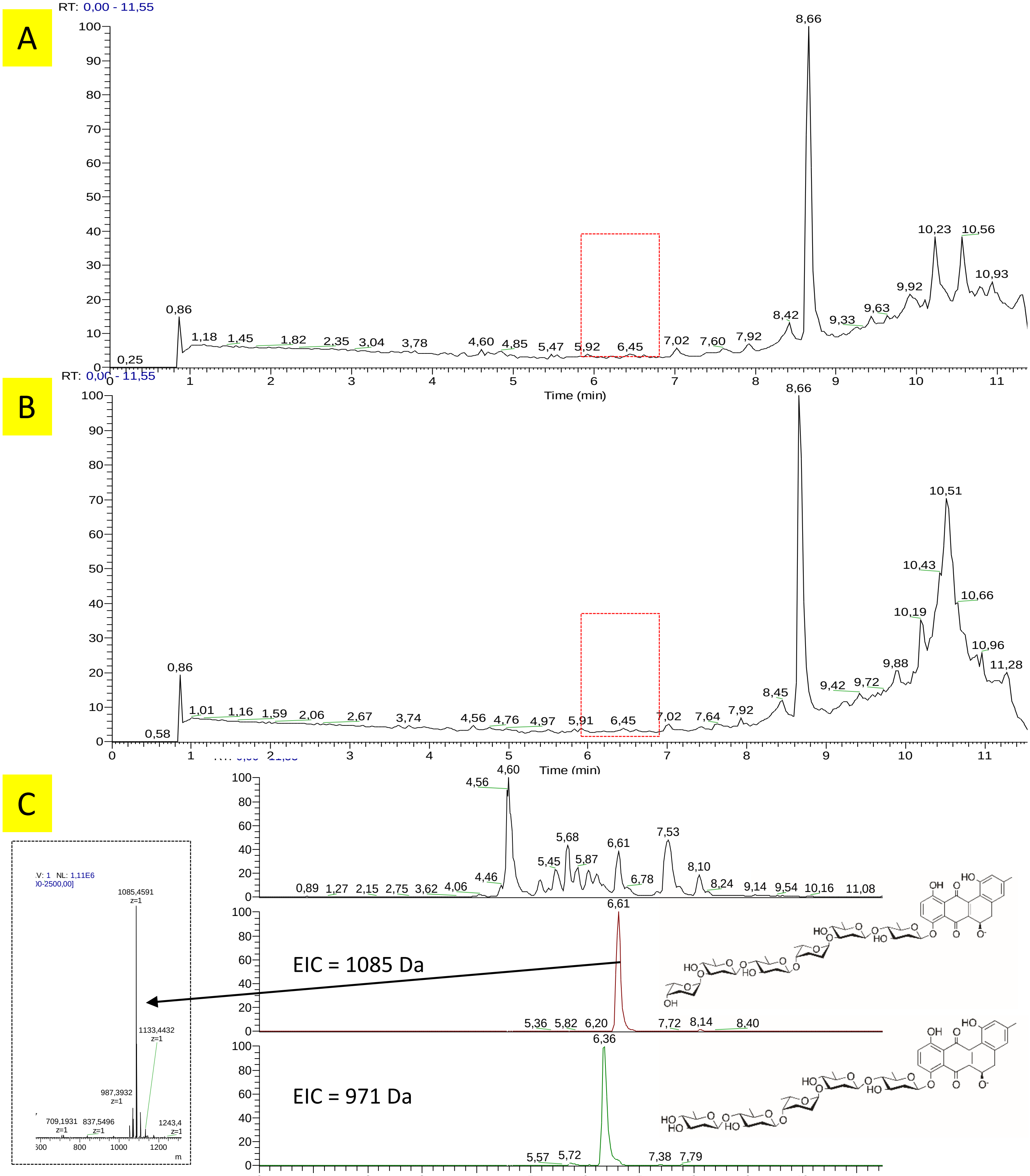

Fig. S4. *S. cyanogenus* S136 and S136 pGM4181$^+$ show qualitatively identical secondary metabolomes in YMPG. Total ion chromatograms (positive ionization) of ethyl-acetate-methanol extracts from the biomass of *S. cyanogenus* S136 (**A**) and S136 pGM4181$^+$ (**B**) grown in YMPG for 120 h. Red dashed rectangle marks the retention time window where lucensomycin and landomycins (La) appear; please see for comparison also Fig. 1 and the TIC traces from YMPG in negative ionization mode (**C**) for S136, where La become detectable. Accumulation of La was increased in S136 pGM4181$^+$ ((1.6±02)×10^4 au) as compared to control strain ((0.7±0.3)×10^4 au); these titers are ~100-fold less than those observed in SG (see Suppl. Ref 2).

Table S2. Lcm activity against different bacterial and fungal species.

Test-culture	Zone of growth inhibition*, mm
Escherichia coli DH5α	No growth inhibition zone
Bacillus cereus	No growth inhibition zone
Staphylococcus aureus	No growth inhibition zone
Debaryomyces hansenii	11±2
Aspergillus niger	16±2
Fusarium oxisporum	24±3

*Paper discs (Whatman 3MM, Ø 5mm) were impregnated with 15 µl of methanol solution containing 10 µg of Lcm
Results represent mean values ± SD of five independent experiments

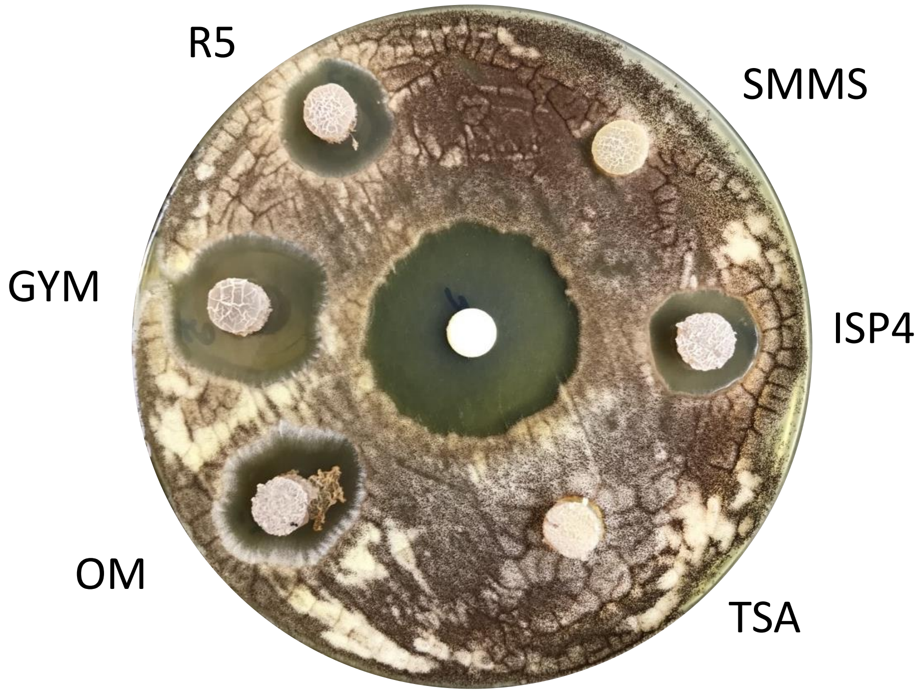

Fig. S5. Agar plugs of *S. cyanogenus* ΔlanI7 pGM4181^{+} exhibit activity against *Fusarium oxisporum*. Strains were grown for 120 h on the agar media and stacked on top of freshly seeded lawn of *F. oxisporum*. In the center of the plate a paper disc carrying 10 µg of Lcm is placed.

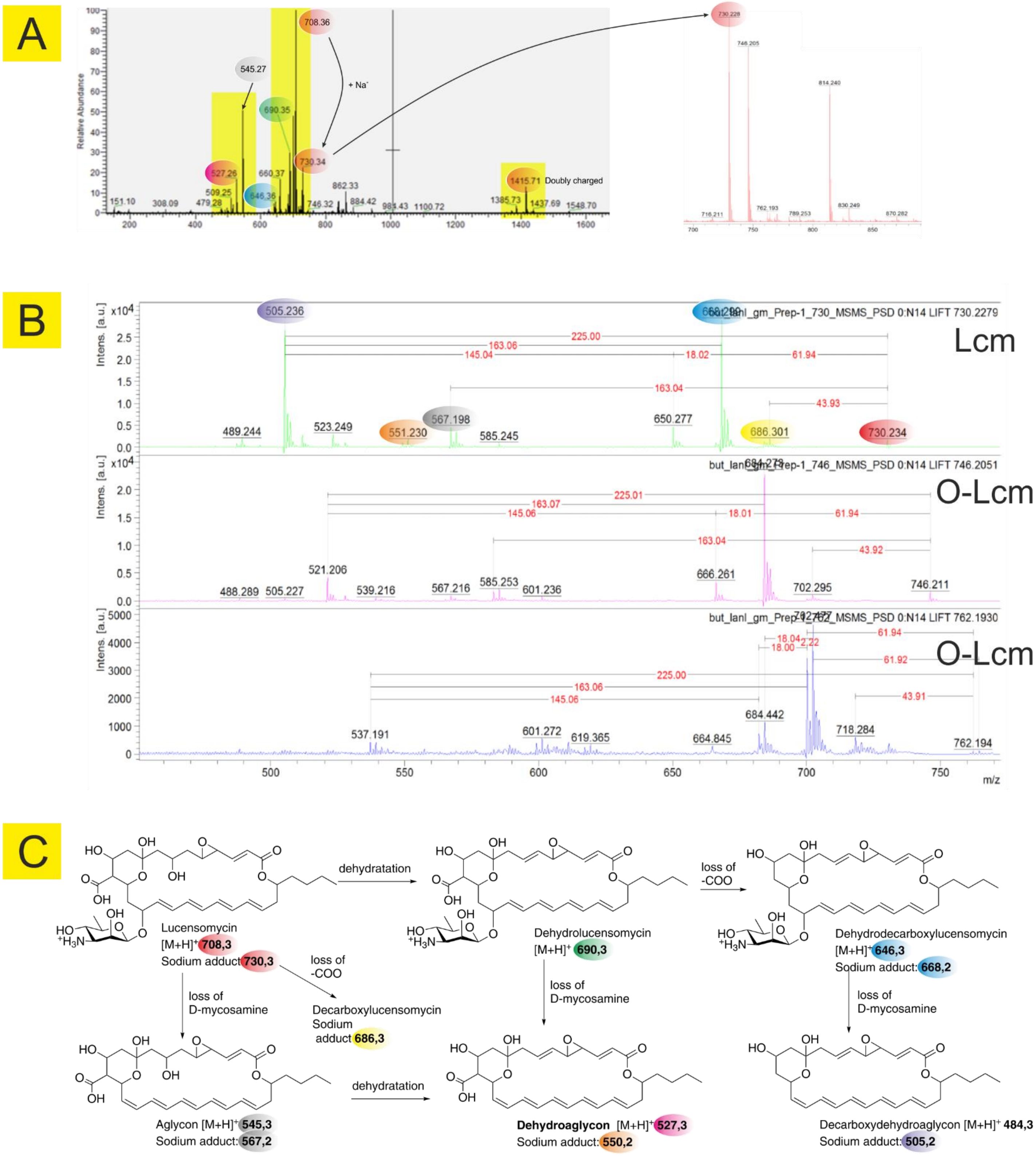

Fig. S6. Fragmentation pattern of 708.35 Da compound is consistent with the chemical structure of Lcm. In-source (+)-ESI fragmentation of the 708.35 Da compound (**A**) and (+)-ESI-MS^2 spectrum for 730.34 Da compound (Na$^+$ adduct of 708.35 Da compound; **B**) are shown; both agree with the CID pathway depicted at the bottom of the figure (**C**). Under MS conditions the dehydration position was arbitrarily chosen. Note that strain accumulated several hydroxylated derivatives of Lcm (O-Lcm; 746.2 Da, 762.2 Da, Na adducts; see **A**, **B**) whose structures could not be inferred due to low yield.

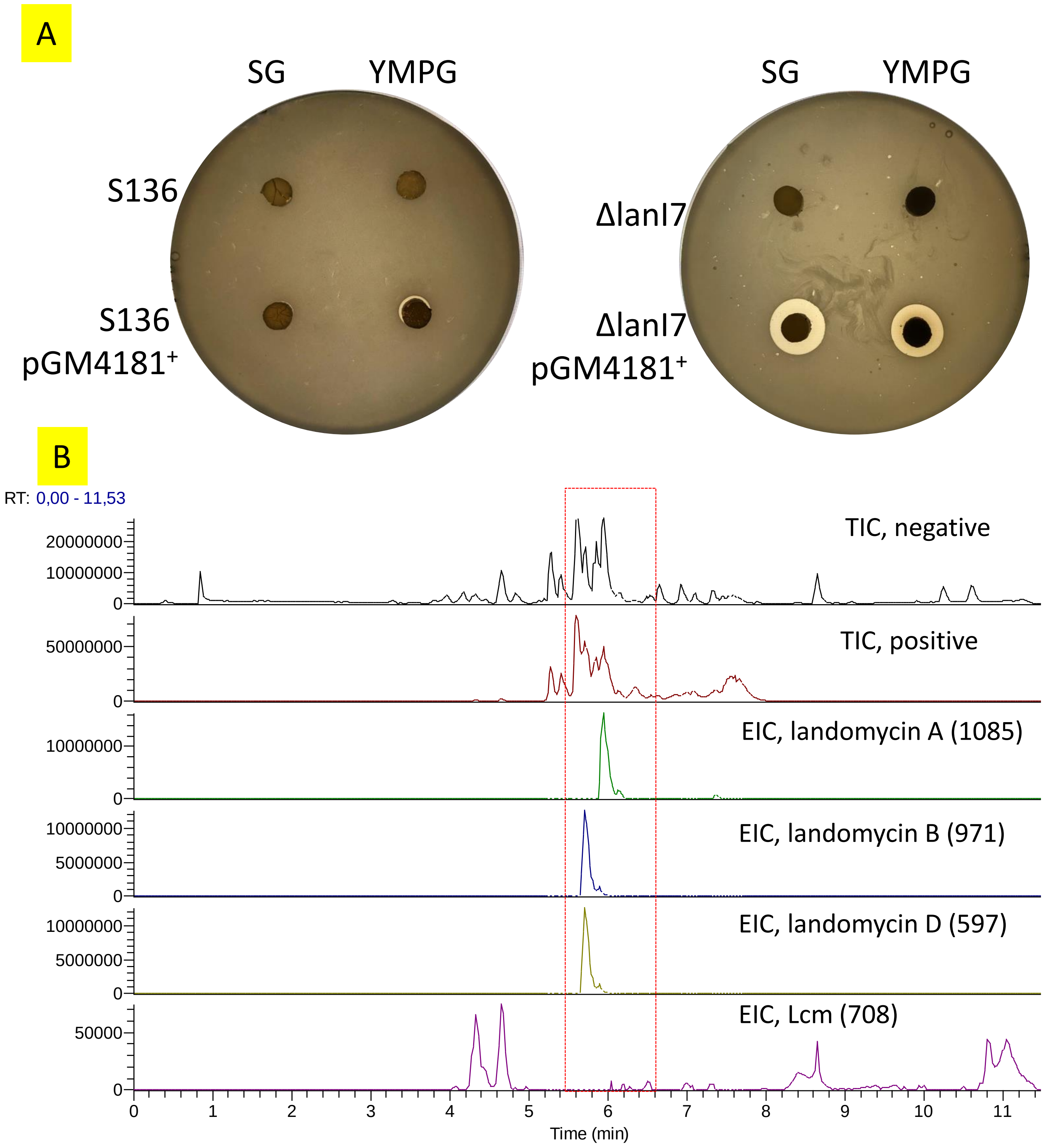

Fig. S7. The AdpA-induced production of Lcm is barely detectable or completely arrested under conditions where landomycin biosynthesis occurs. (**A**) Agar plug assay of *S. cyanogenus* S136 and S136 pGM4181^{+} strain grown on SG and YMPG agars against *D. hansenii*. Strains were grown for 120 h. Photos represent typical result of four biological replicates. (**B**) LC-MS traces of methanol-acetone extracts of the biomass of *S. cyanogenus* S136 pGM4181^{+} strain grown for 72 h in SG. Extracted ion chromatograms (EIC) show the presence of landomycins A, B and D, and absence of Lcm. See also Fig. S4 for landomycin structures. Red dashed rectangle marks the retention time window where the analyzed compounds occur.

A

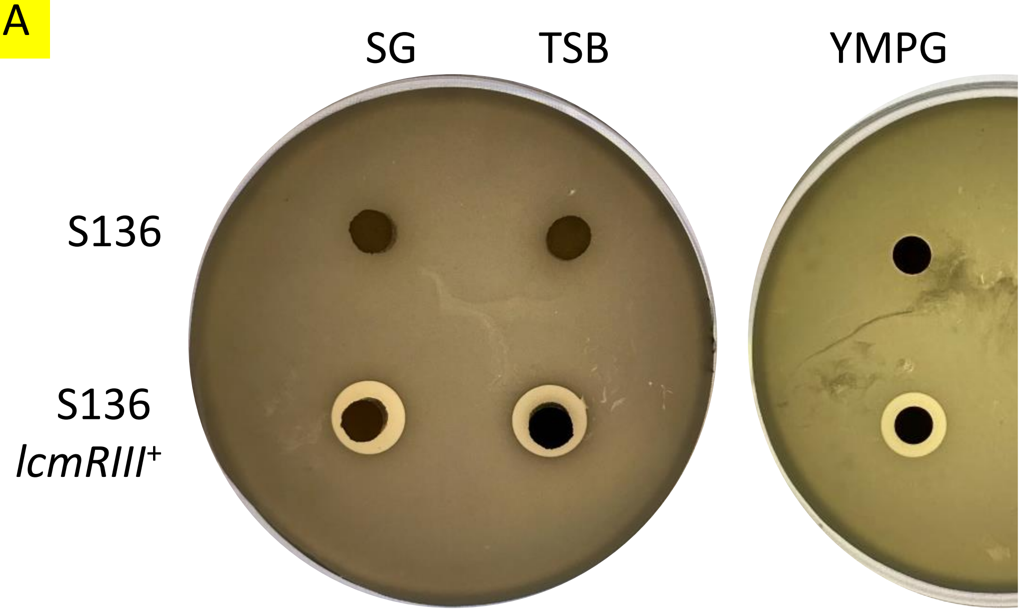

B

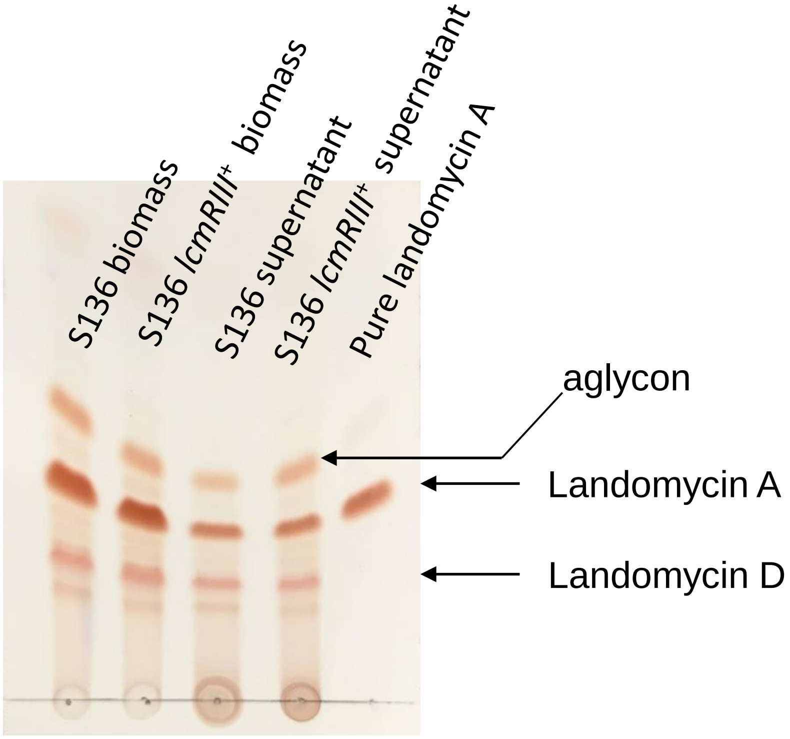

Fig. S8. Pathway-specific regulatory gene *lcmRIII* under control of constitutive promoter *ermEp* induces Lcm production in AdpA- and medium-independent manner while leaving landomycin production unperturbed. (**A**) Strains and media are labeled around the photo. Agar plugs for the bioassay were taken from lawns grown for 120 h. (**B**). TLC of landomycins extracted separately from biomass and spent medium (5-mL samples) of the strains labeled on the photo above. Differences in the biomass (dry weight) did not exceed 10% of the mean value. The TLC image represents typical result of six biological replicates.

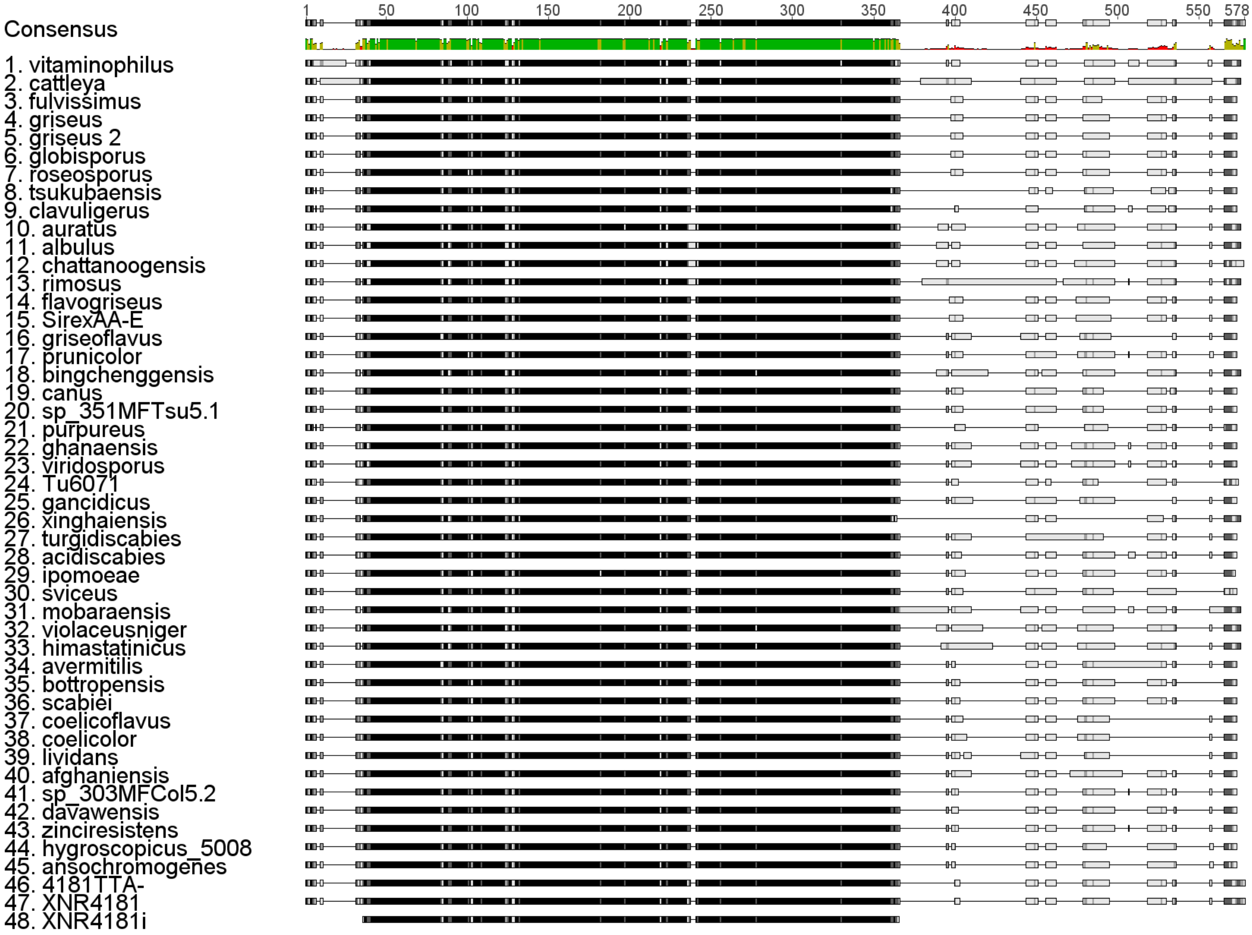

Fig. S9. Multiple amino acid sequence alignment constructed to discover the most conserved region of AdpA proteins (Geneious alignment). Novel AdpA variants constructed in this work – 4181$_{\text{TTA-}}$ *(*XNR_4181$_{\text{TTA-}}$*)*, XNR4181i *(*XNR_4181i*)* – together with *S. albus adpA (*XNR_4181*)* – are shown at the bottom of the alignment.

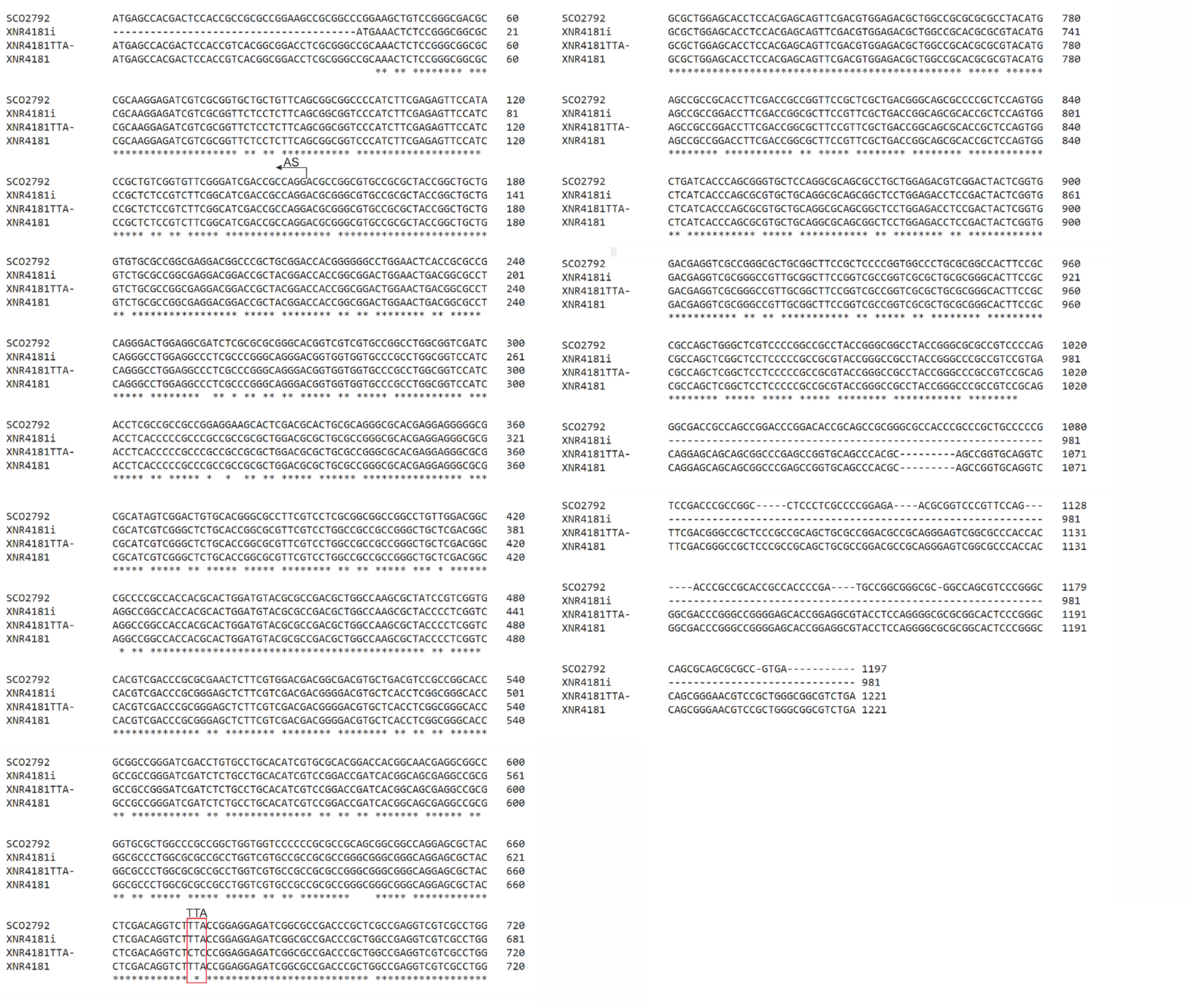

Fig. S10. Multiple nucleotide sequence alignment of *adpA*$_{sco}$, *XNR_4181* and the new alleles of the latter. Positions of *adpA*$_{sco}$ antisense RNA transcription start point (AS) and rare TTA codon are highlighted.

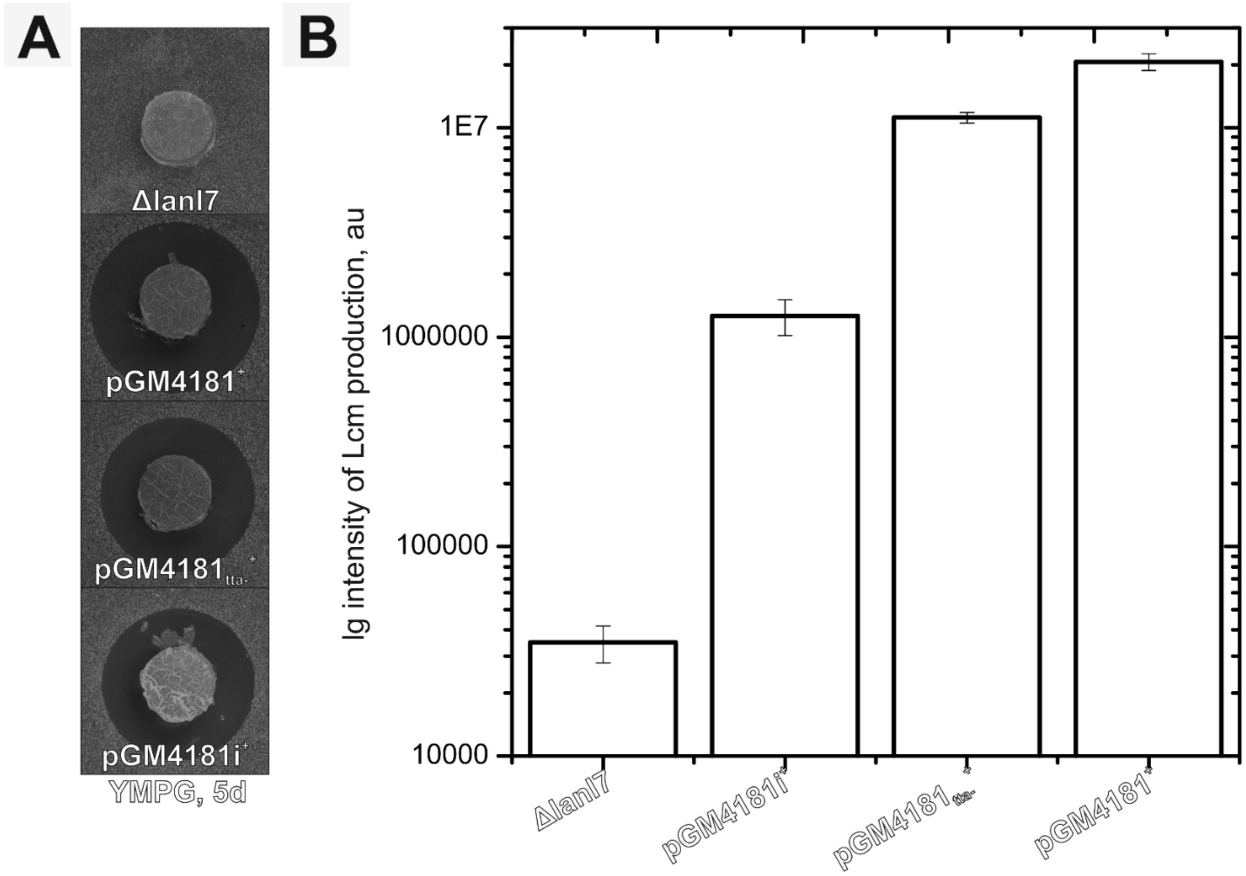

Fig. S11. Novel *adpA* alleles activate Lcm production by *S. cyanogenus* ΔlanI7, as judged from agar plug assay (**A**) and LC-MS results (**B**). Areas of Lcm mass peaks (708.35 Da (M+H)$^+$) were integrated and represented as arbitrary units (au); please note logarithmic scale of the *y*-axis. Data represent mean values of three independent experiments ± 2SD.

Supplementary References

1. Kieser, T., Bibb, M. J., Buttner, M. J., Chater, K. F. & Hopwood D. A. Practical *Streptomyces* genetics (Norwich: John Innes Foundation, 2000).
2. Yushchuk, O. *et al.* Heterologous AdpA transcription factors enhance landomycin production in *Streptomyces cyanogenus* S136 under a broad range of growth conditions. *Appl. Microbiol. Biotechnol*. **102**, 8419-8428 (2018). doi: 10.1007/s00253-018-9249-1.
3. Rebets, Y. *et al.* Function of *lanI* in regulation of landomycin A biosynthesis in *Streptomyces cyanogenus* S136 and cross-complementation studies with *Streptomyces* antibiotic regulatory proteins encoding genes. *Arch. Microbiol.* **189,** 111-120 (2008). doi: 10.1007/s00203-007-0299-5.
4. Herrmann, S. *et al.* Site-specific recombination strategies for engineering actinomycete genomes. *Appl. Environ. Microbiol.* **78**, 1804-12 (2012). doi: 10.1128/AEM.06054-11.
5. Makitrynskyy, R. *et al.* Pleiotropic regulatory genes *bldA*, *adpA* and *absB* are implicated in production of phosphoglycolipid antibiotic moenomycin. *Open Biol.* **3**, 130121 (2013). doi: 10.1098/rsob.130121.
6. Koshla, O. *et al.* Gene *miaA* for post-transcriptional modification of $tRNA_{XXA}$ is important for morphological and metabolic differentiation in *Streptomyces*. *Mol. Microbiol*. **112**, 249-265 (2019). doi:10.1111/mmi.14266.

.

Original versions of photos from main text Fig. 1.

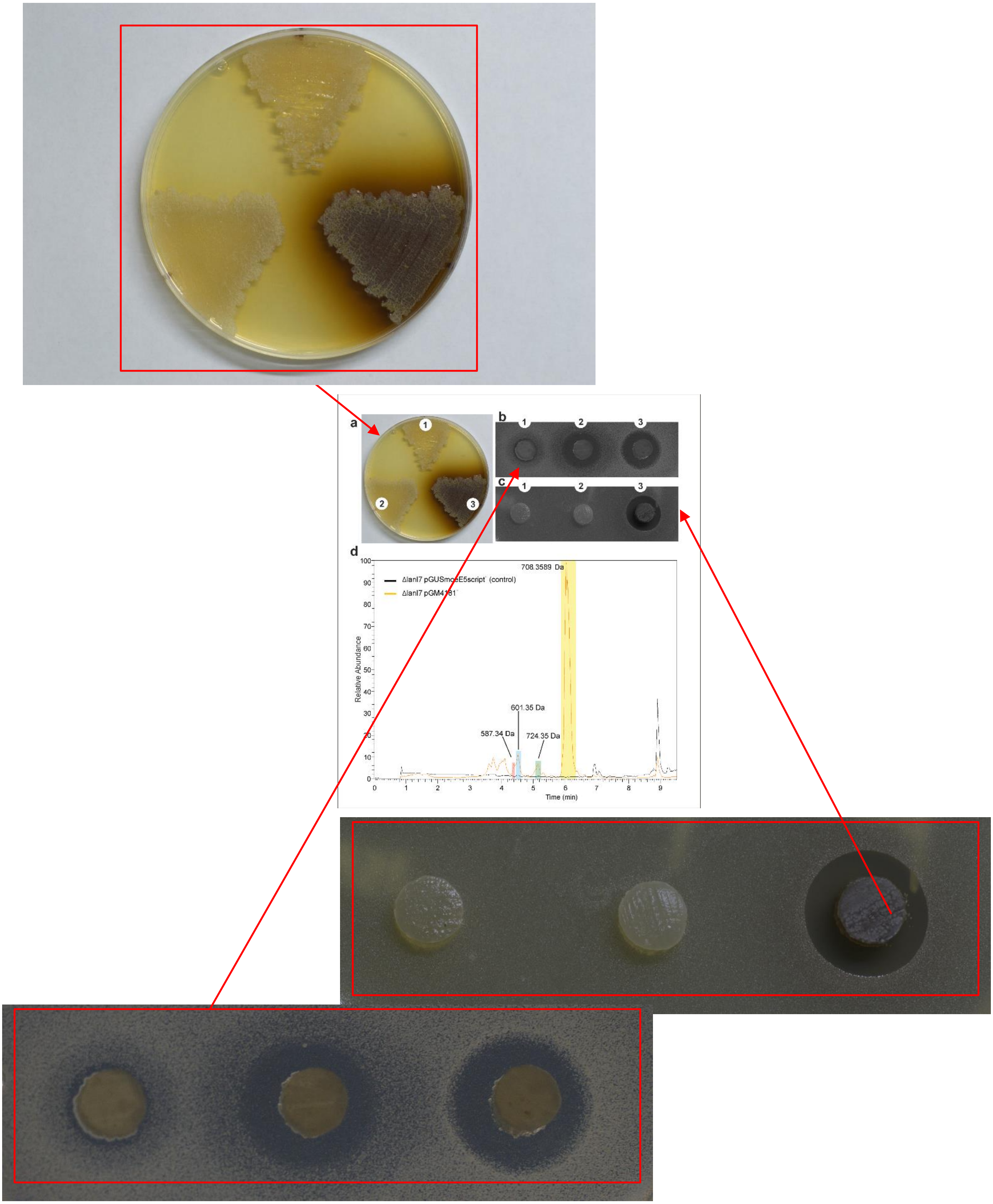

Original versions of photos from main text Fig. 2.

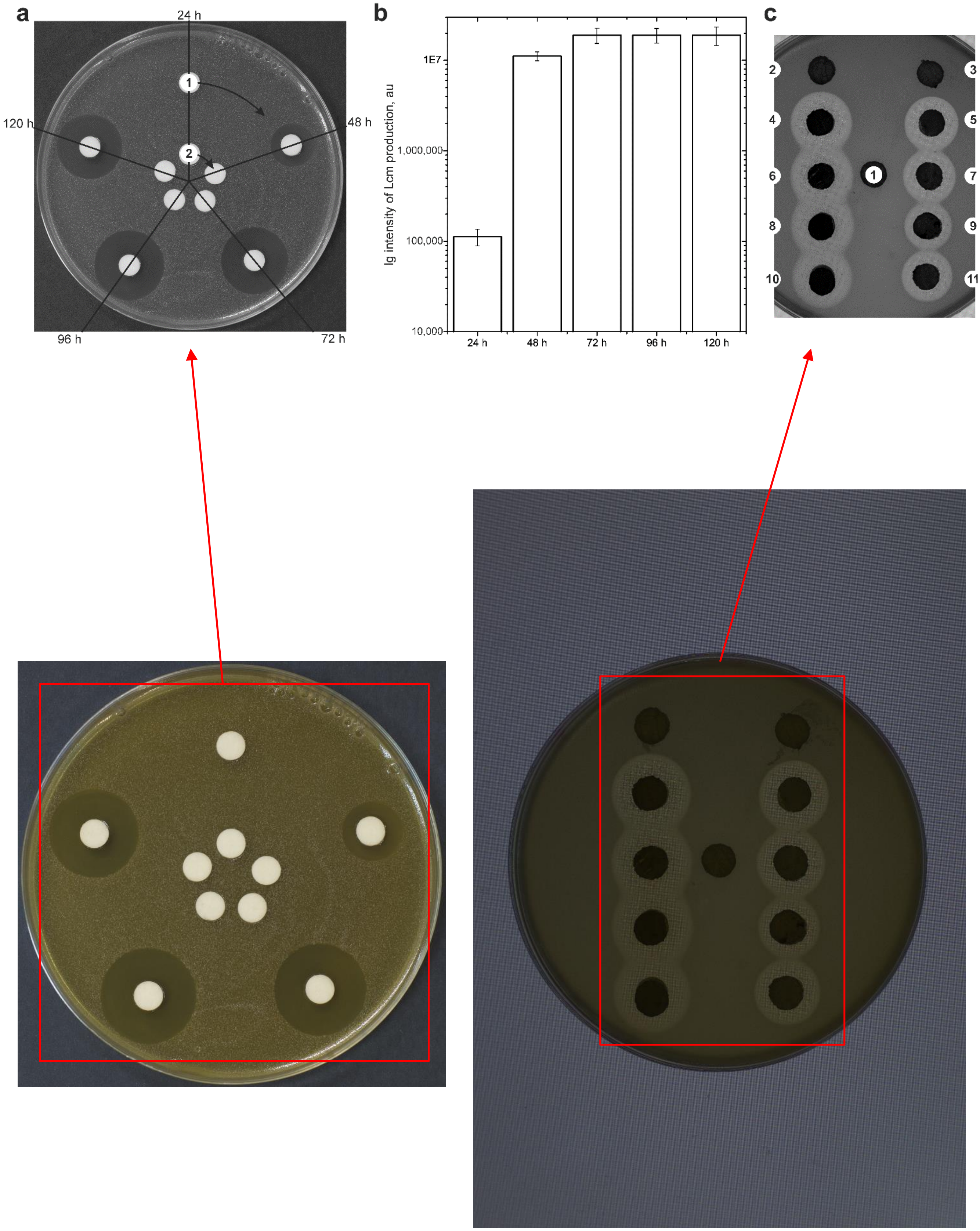

Original version of Fig. 3

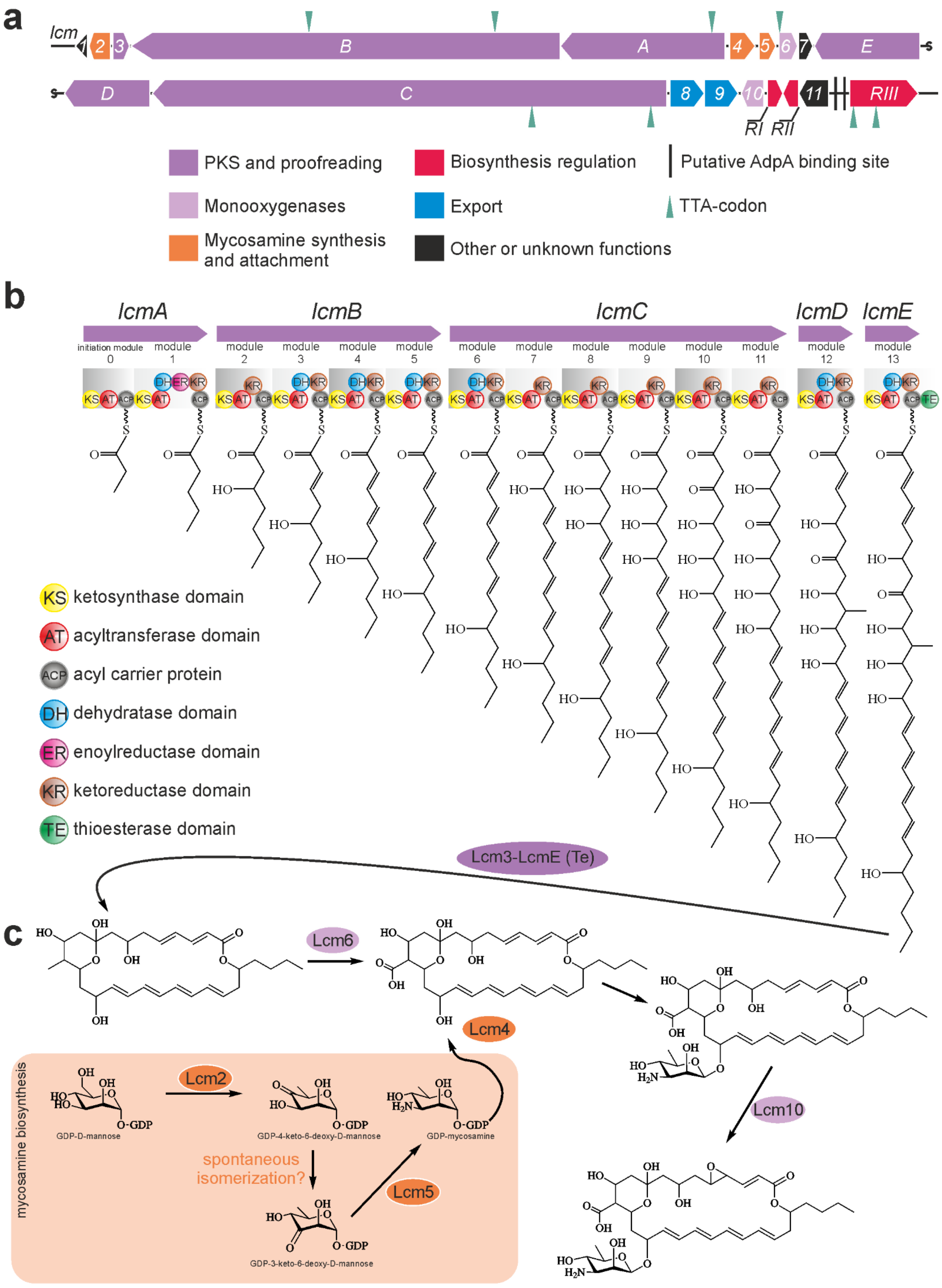

Original versions of photos from main text Fig. 4

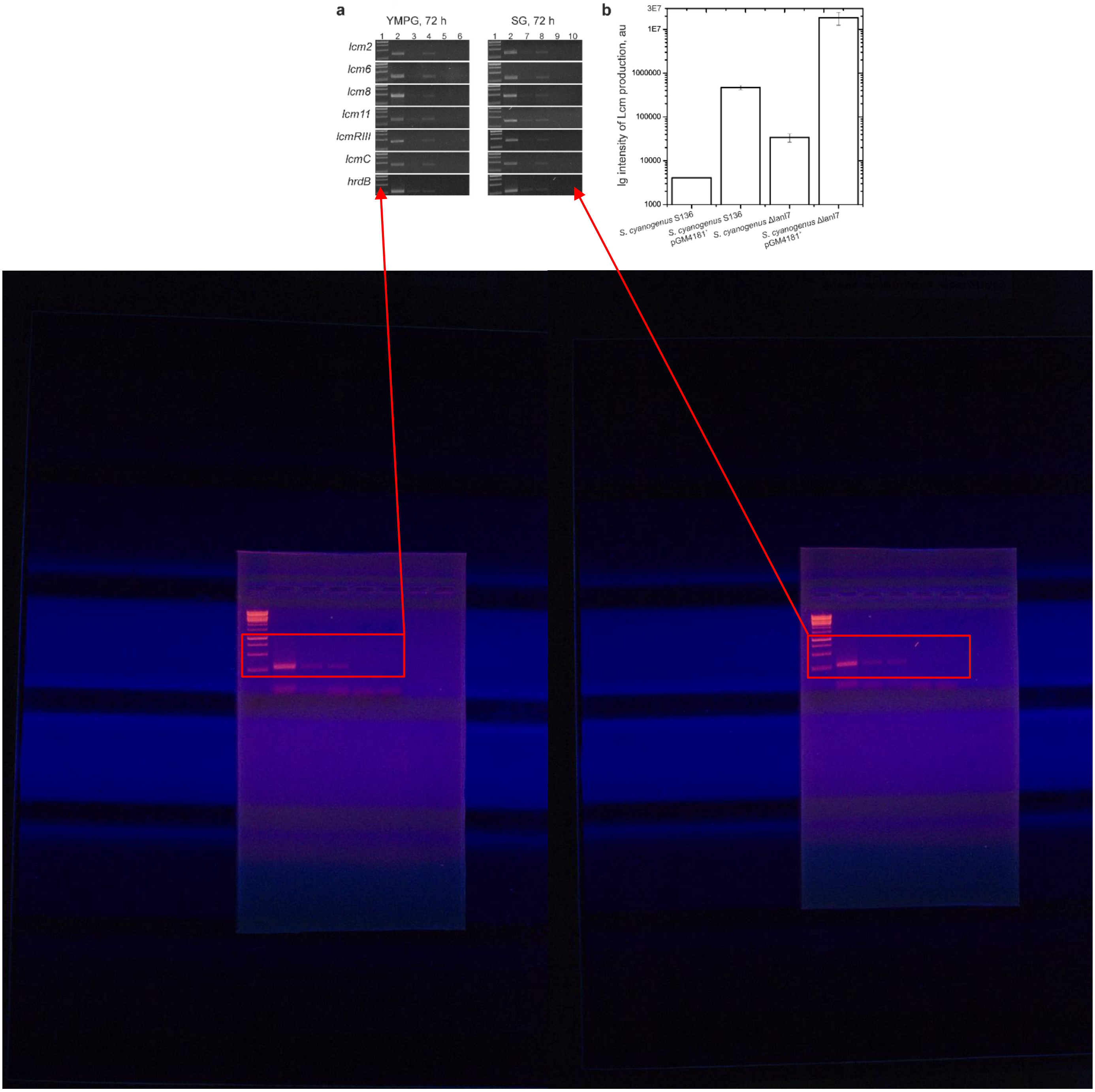

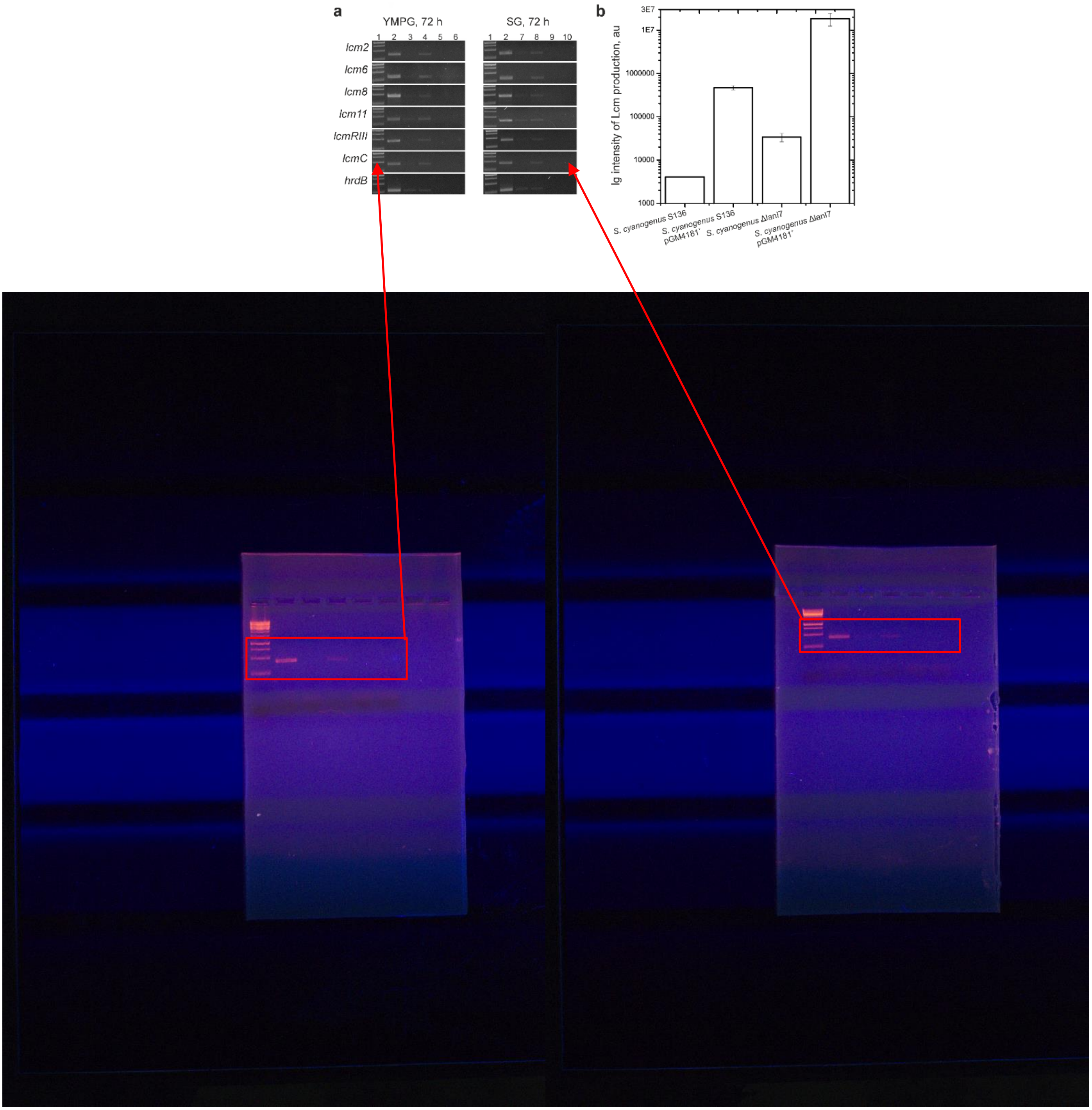

a
YMPG, 72 h
SG, 72 h
1 2 3 4 5 6
1 2 7 8 9 10
lcm2
lcm6
lcm8
lcm11
lcmRIII
lcmC
hrdB
b
lg intensity of Lcm production, au
3E7
1E7
1000000
100000
10000
1000
S. cyanogenus S136
S. cyanogenus S136 pGM4181+
S. cyanogenus ΔlanI7
S. cyanogenus ΔlanI7 pGM4181+

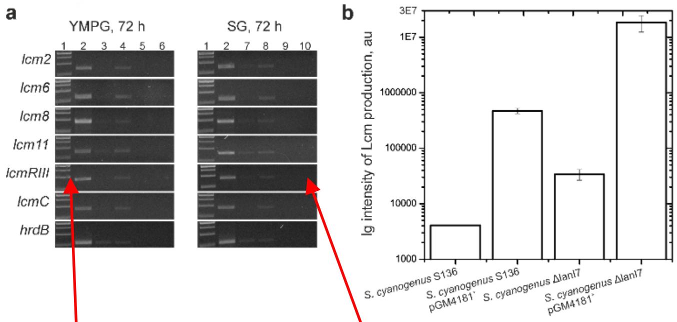
a
YMPG, 72 h
SG, 72 h
lcm2
lcm6
lcm8
lcm11
lcmRIII
lcmC
hrdB
b
lg intensity of Lcm production, au
S. cyanogenus S136
S. cyanogenus S136 pGM4181⁺
S. cyanogenus ΔlanI7
S. cyanogenus ΔlanI7 pGM4181⁺

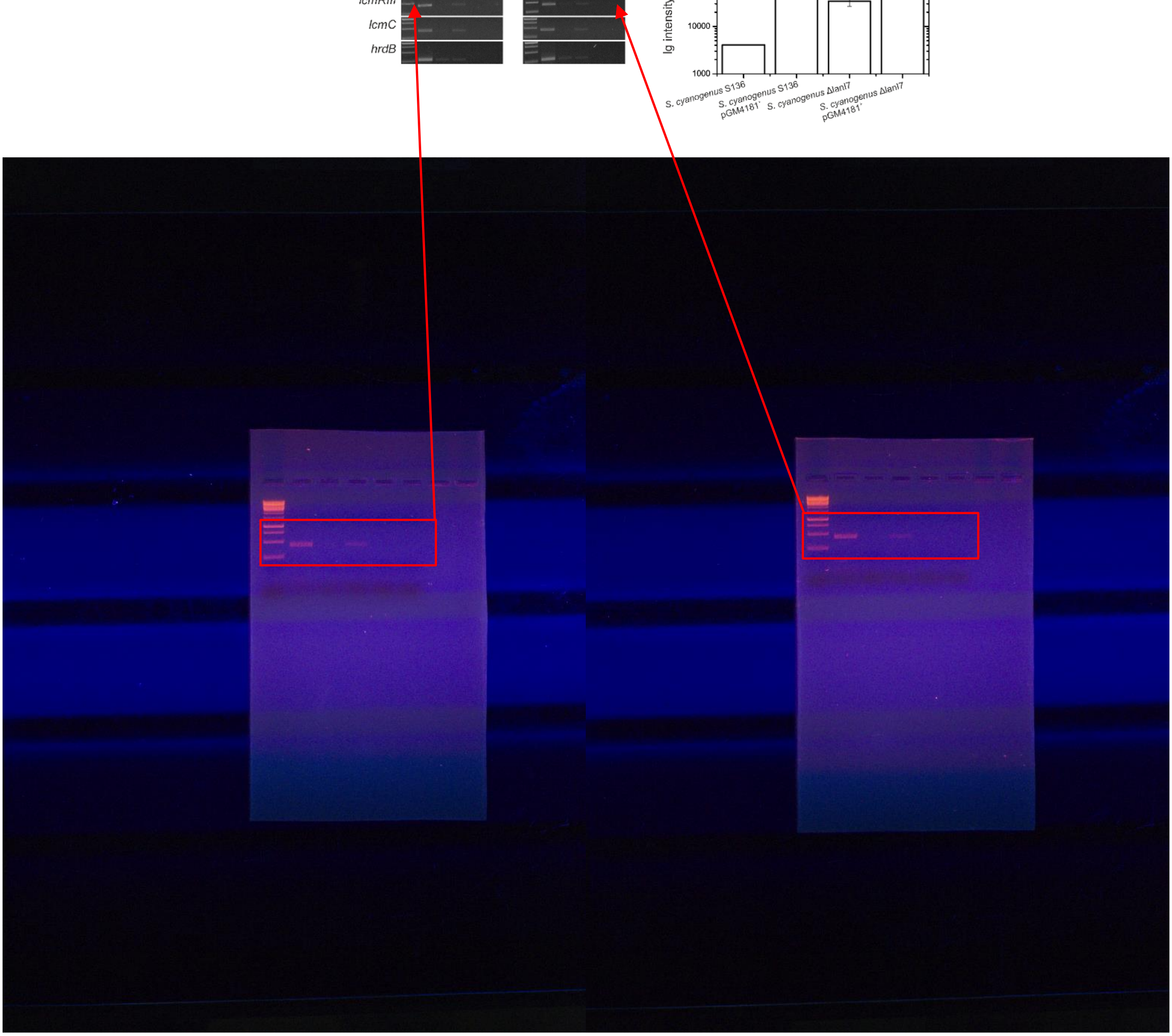

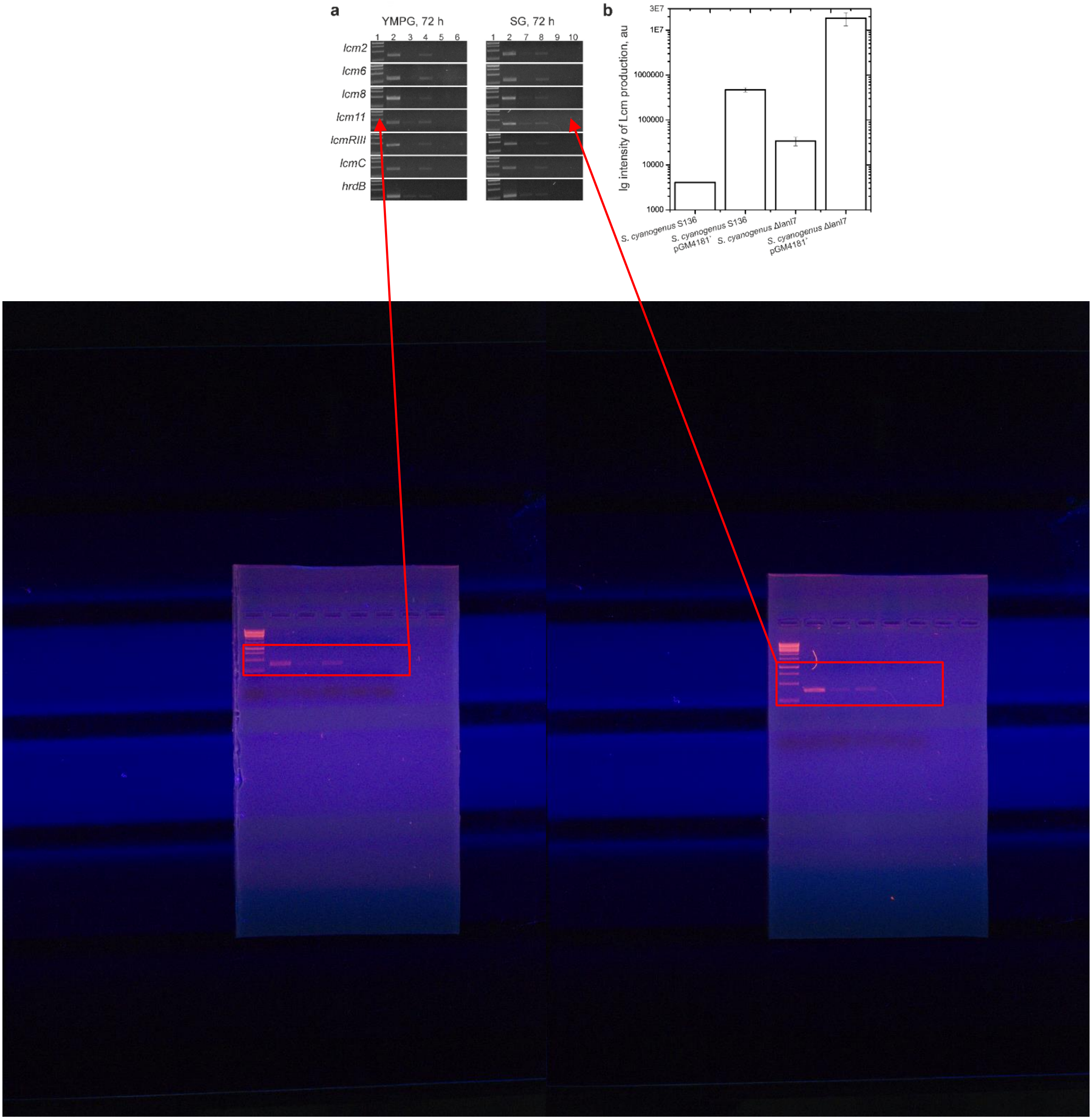
a
YMPG, 72 h
SG, 72 h
1 2 3 4 5 6
1 2 7 8 9 10
lcm2
lcm6
lcm8
lcm11
lcmRIII
lcmC
hrdB
b
lg intensity of Lcm production, au
3E7
1E7
1000000
100000
10000
1000
S. cyanogenus S136
S. cyanogenus S136 pGM4181⁺
S. cyanogenus ΔlanI7
S. cyanogenus ΔlanI7 pGM4181⁺

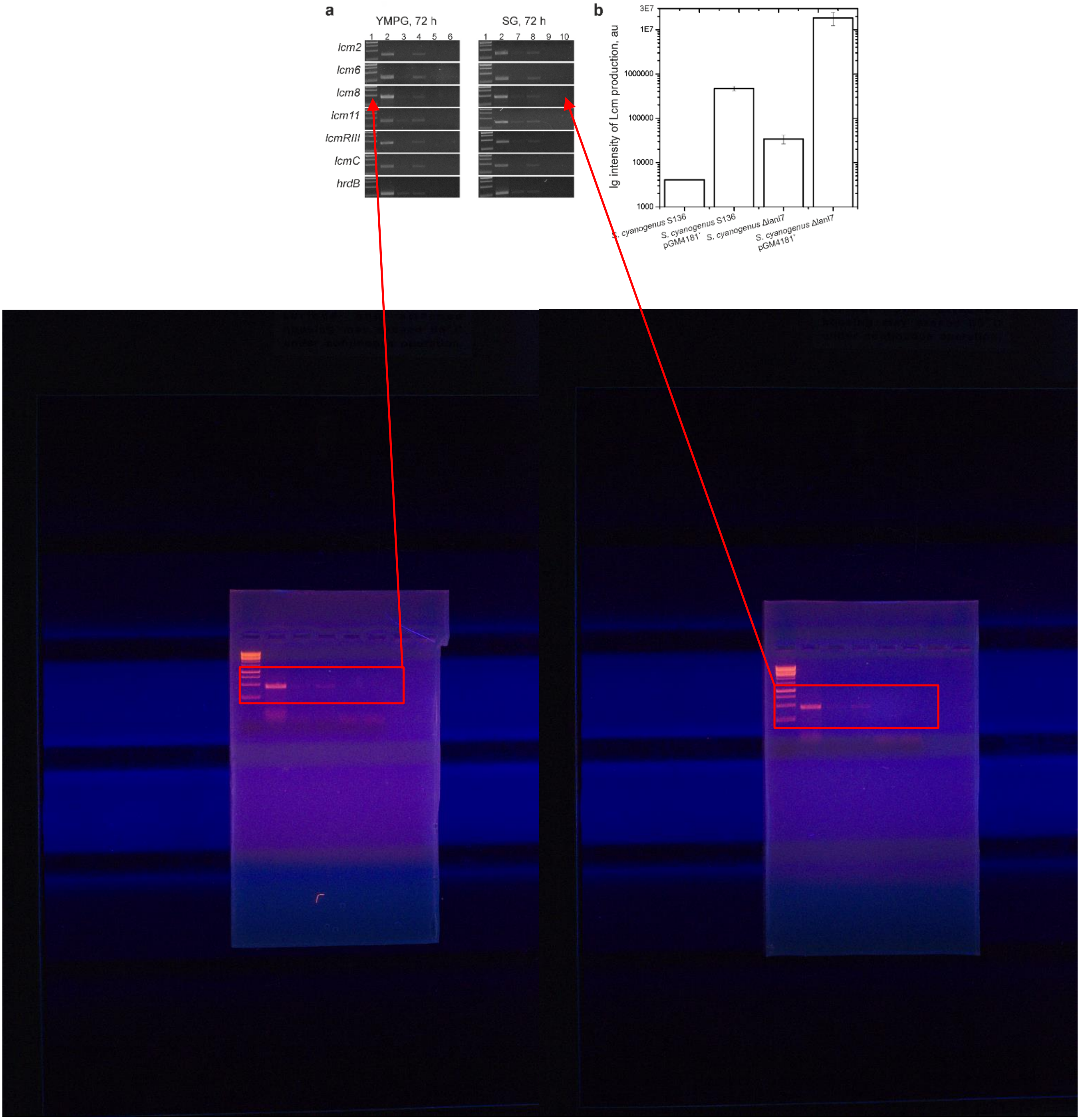
a
YMPG, 72 h
SG, 72 h
1 2 3 4 5 6
1 2 7 8 9 10
lcm2
lcm6
lcm8
lcm11
lcmRIII
lcmC
hrdB
b
Ig intensity of Lcm production, au
3E7
1E7
1000000
100000
10000
1000
S. cyanogenus S136
S. cyanogenus S136 pGM4181⁺
S. cyanogenus ΔlanI7
S. cyanogenus ΔlanI7 pGM4181⁺

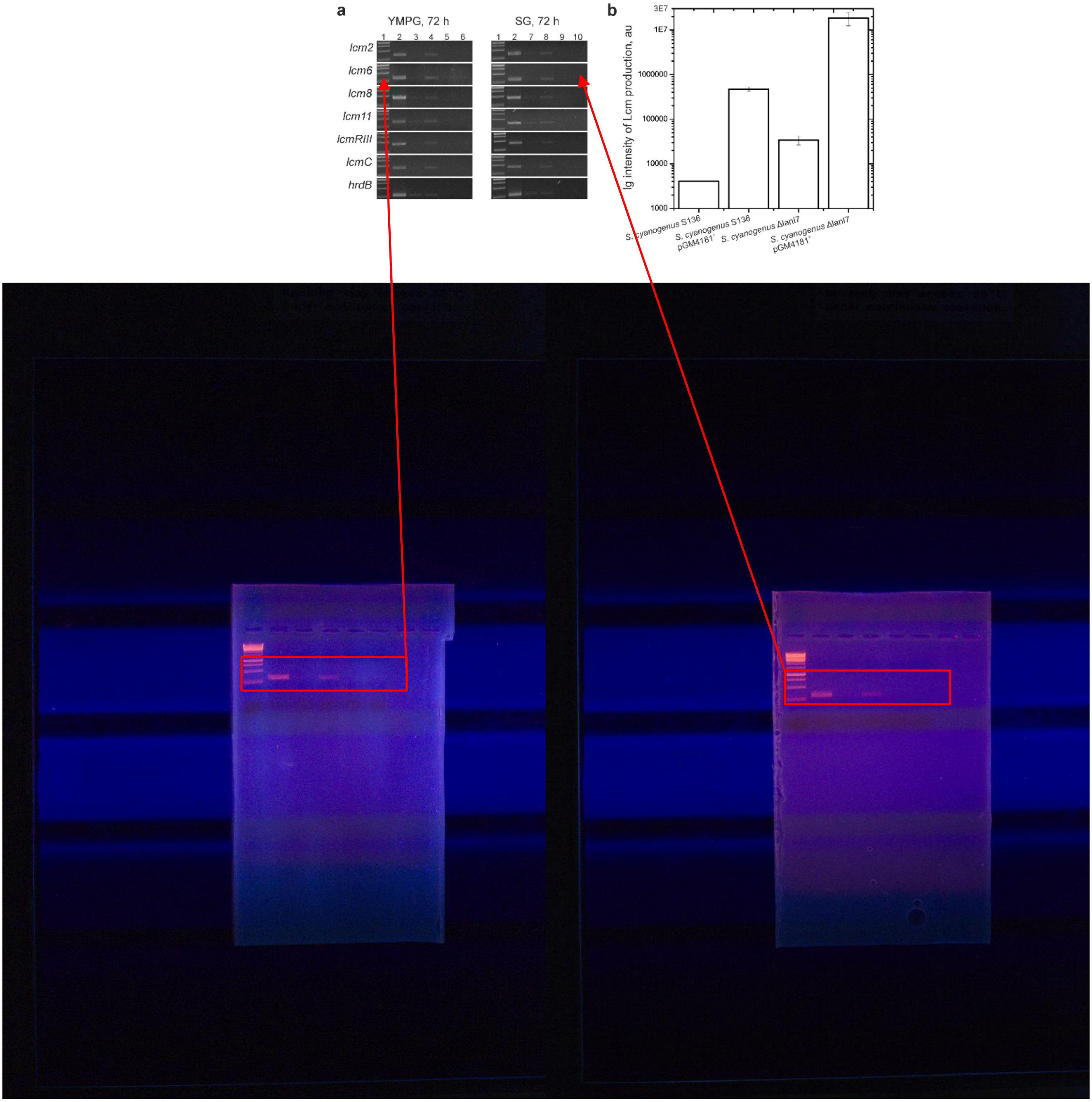
a
YMPG, 72 h
SG, 72 h
1 2 3 4 5 6
1 2 7 8 9 10
lcm2
lcm6
lcm8
lcm11
lcmRIII
lcmC
hrdB
b
lg intensity of Lcm production, au
3E7
1E7
1000000
100000
10000
1000
S. cyanogenus S136
S. cyanogenus S136 pGM4181⁺
S. cyanogenus ΔlanI7
S. cyanogenus ΔlanI7 pGM4181⁺

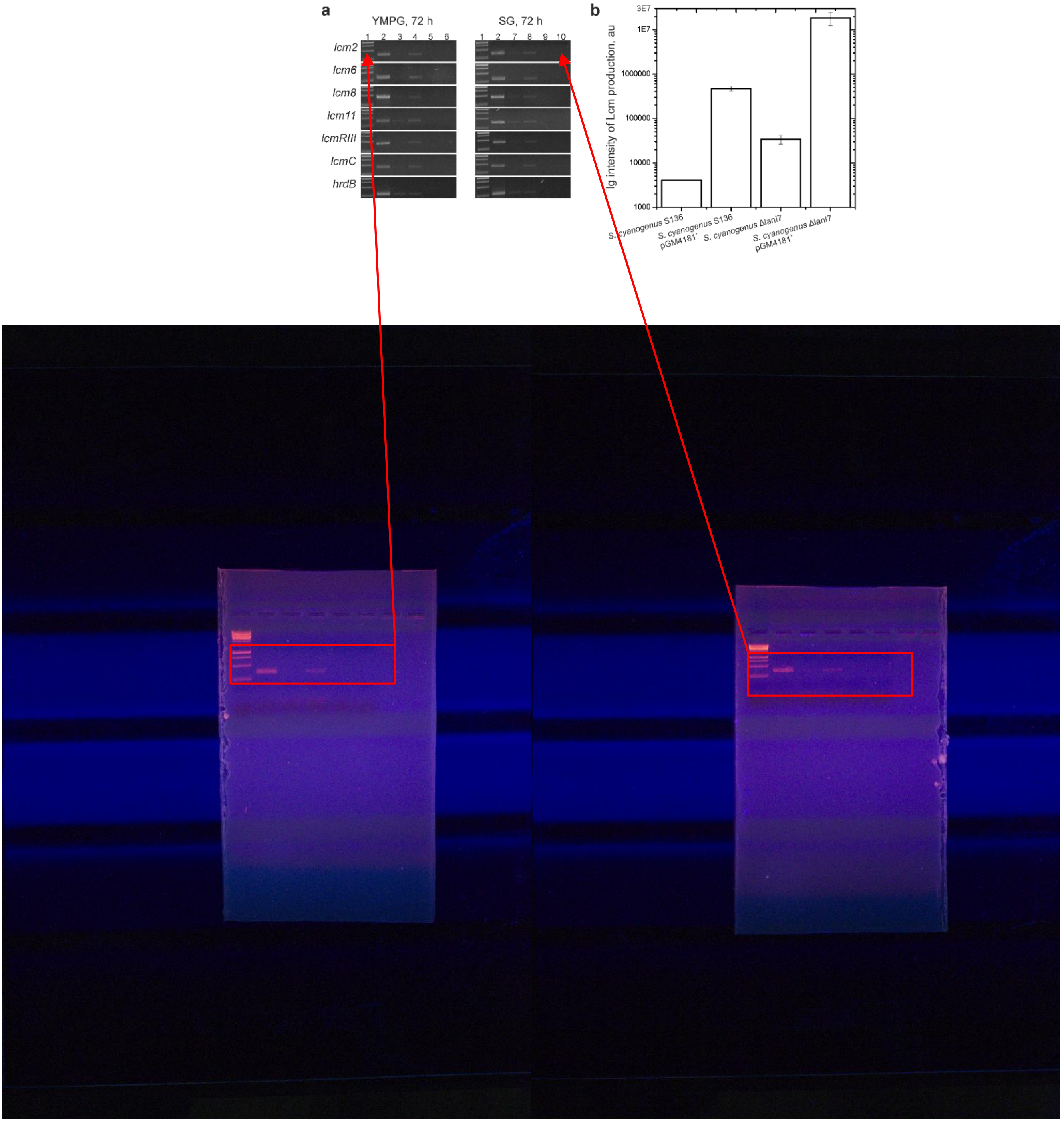
a
YMPG, 72 h
1 2 3 4 5 6
SG, 72 h
1 2 7 8 9 10
lcm2
lcm6
lcm8
lcm11
lcmRIII
lcmC
hrdB
b
lg intensity of Lcm production, au
3E7
1E7
1000000
100000
10000
1000
S. cyanogenus S136
S. cyanogenus S136 pGM4181⁺
S. cyanogenus Δlanl7
S. cyanogenus Δlanl7 pGM4181⁺

Original versions of photos from main text Fig. 5.

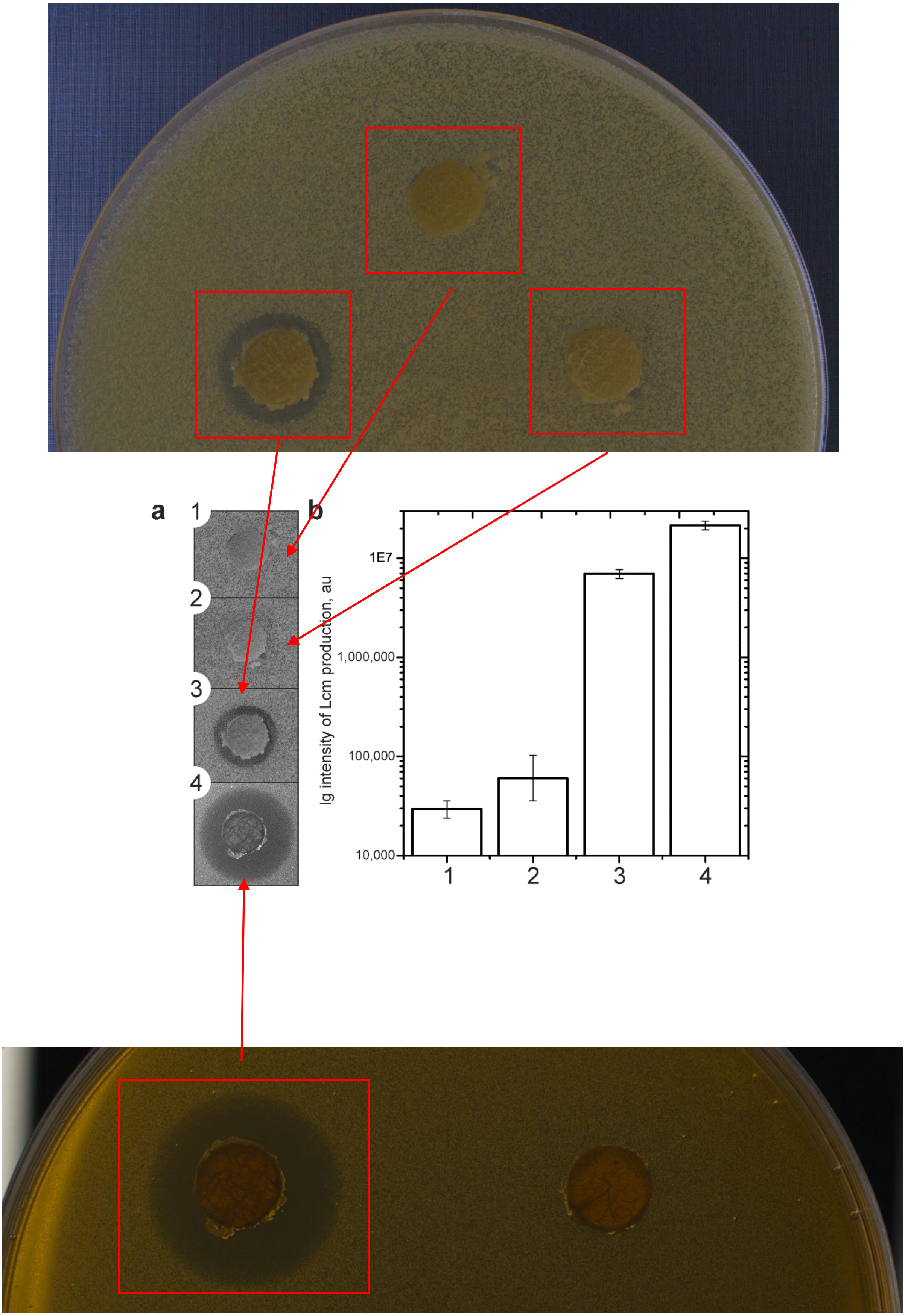

Original version of Fig. 6.

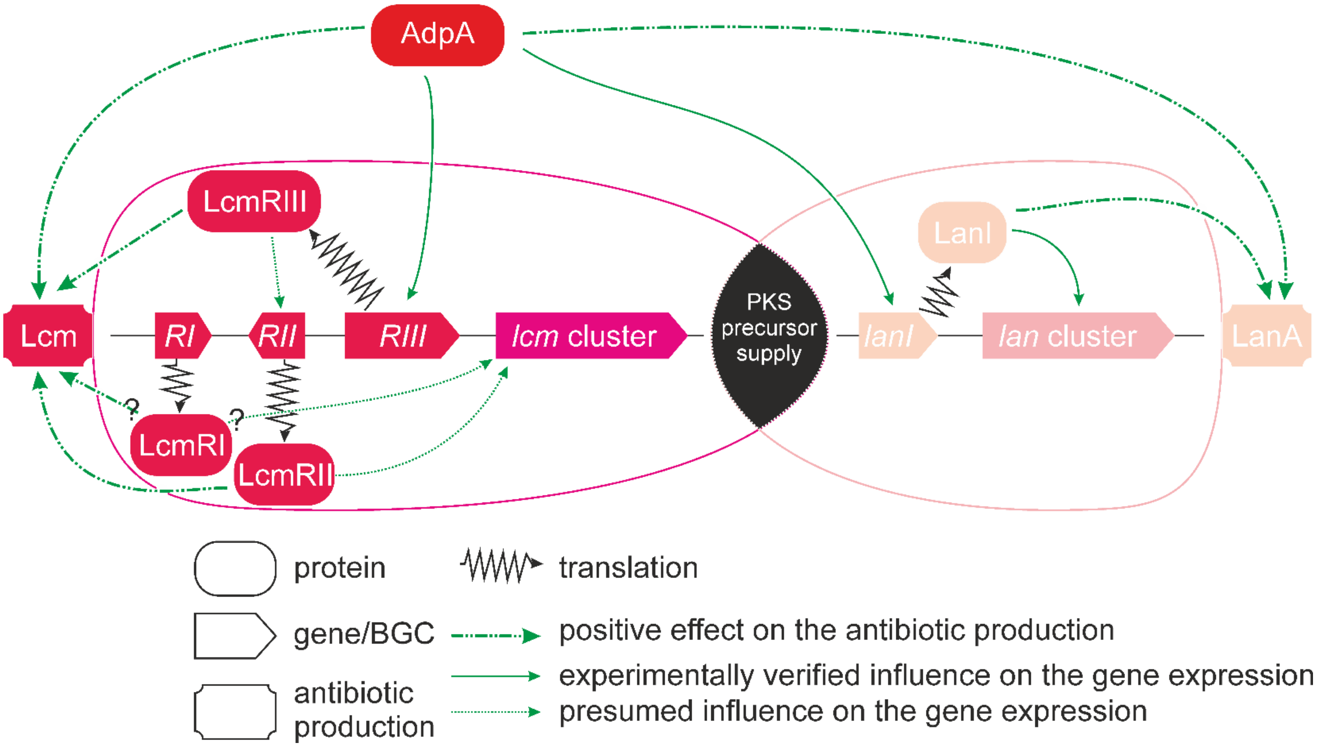

Original version of the agarose gel shown in Fig. S2

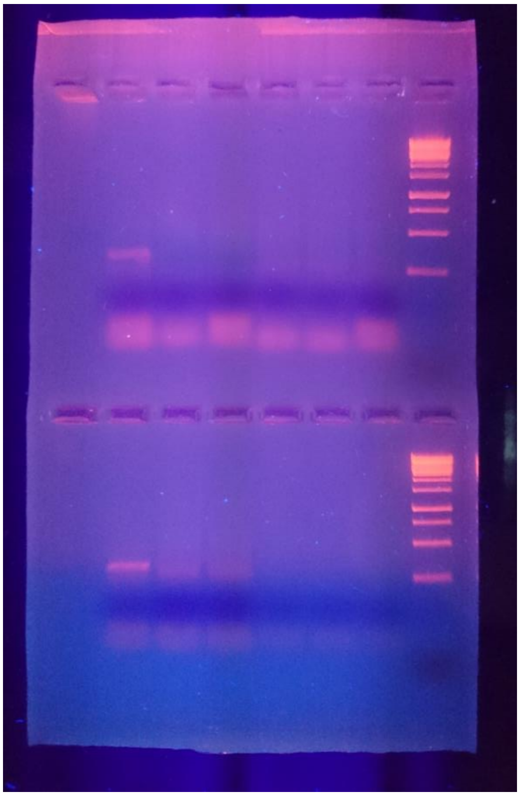

Original versions of the photos from ESM Fig. S3

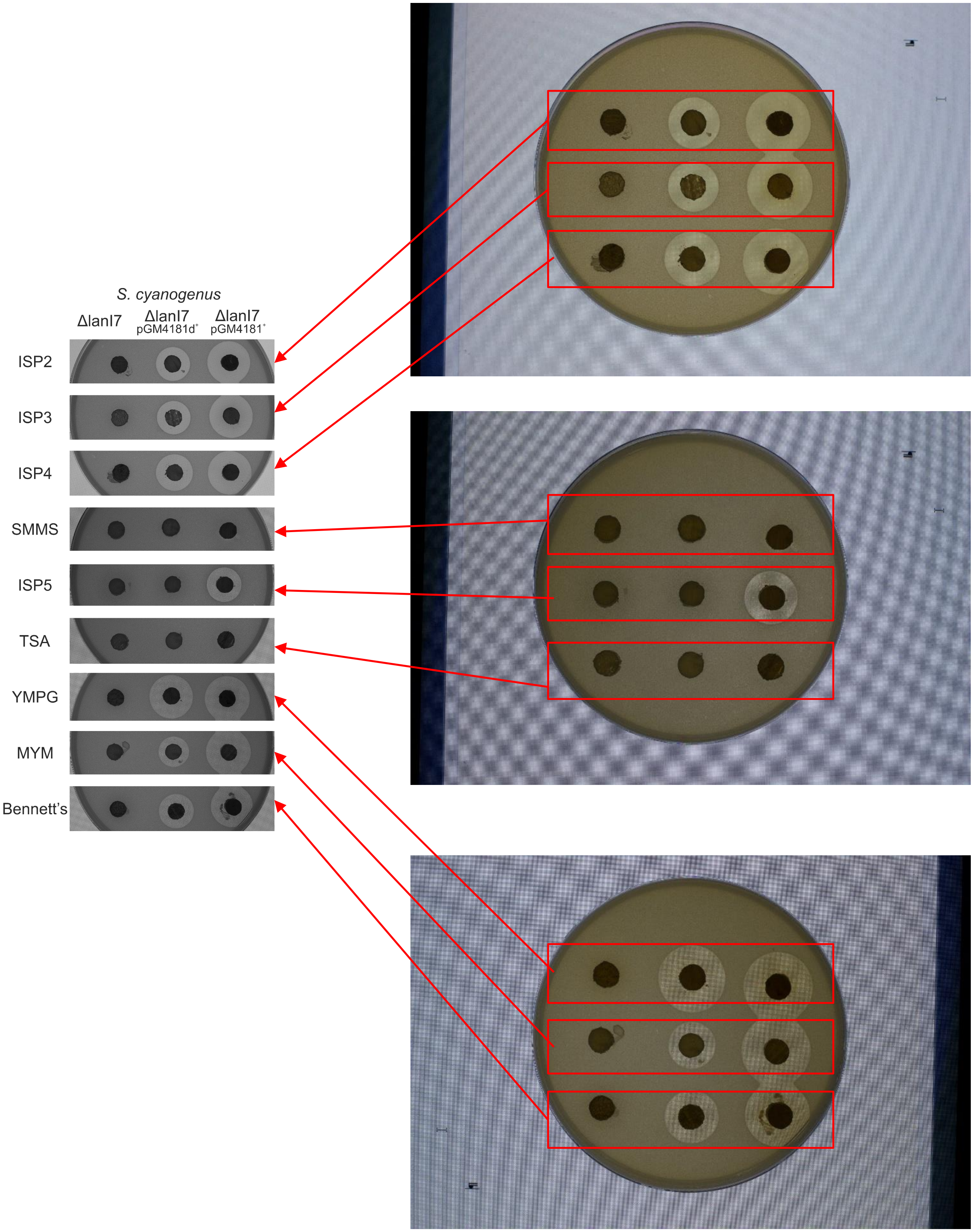

Original versions photos of Petri dishes shown in Fig. S5

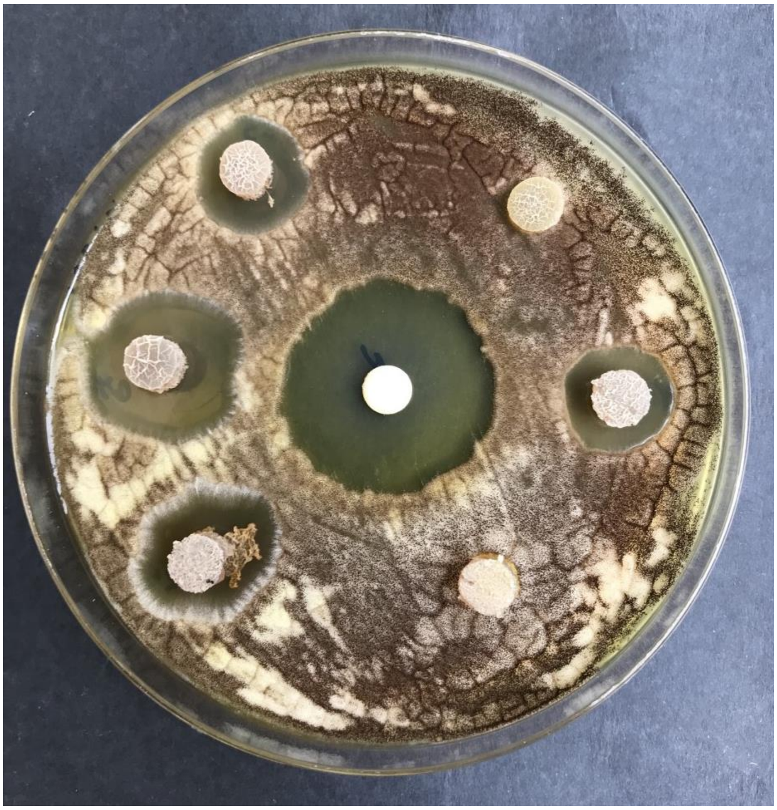

Original versions photos of Petri dishes shown in Fig. S7a

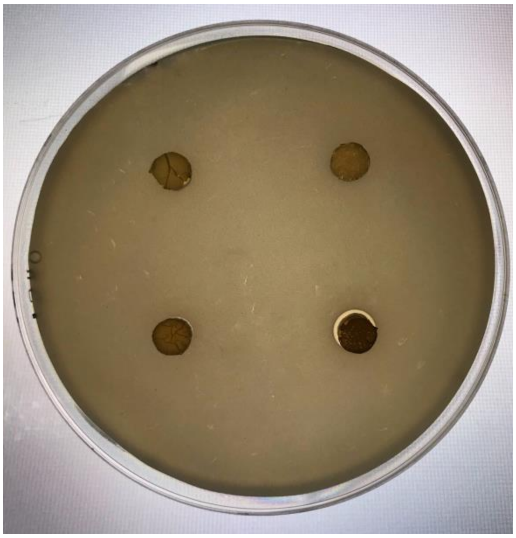

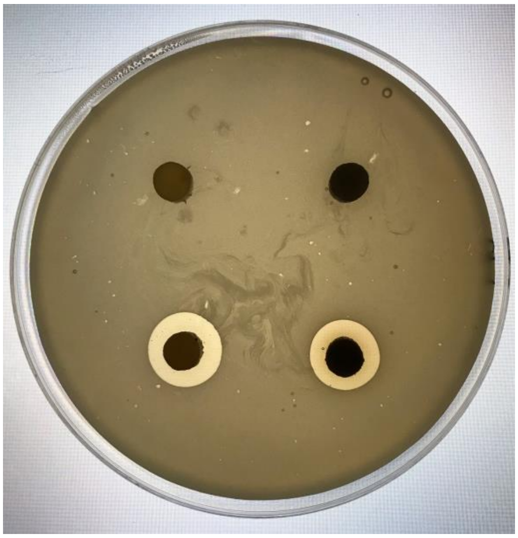

Original versions photos of Petri dishes shown in Fig. S8a

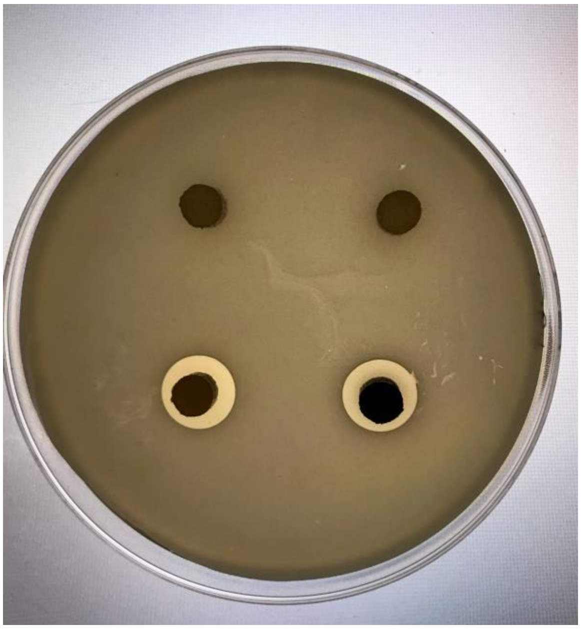

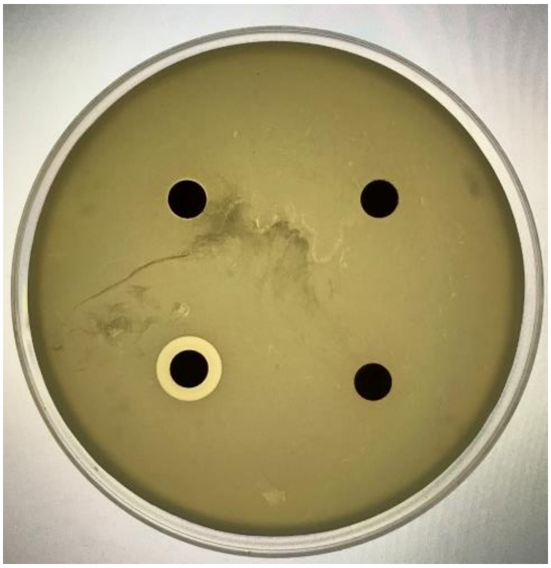

Original versions of the photo from ESM Fig. S11

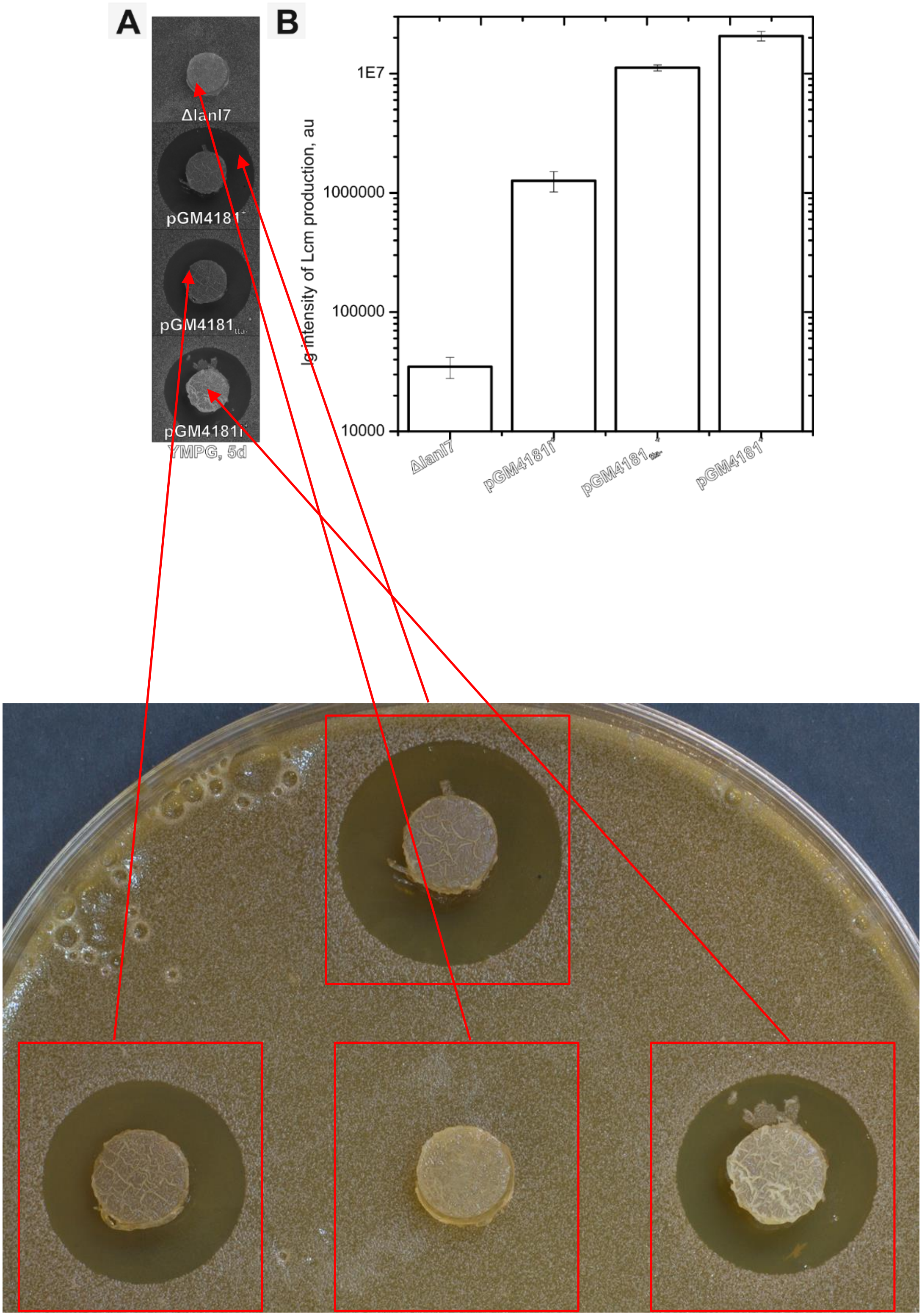

Supplement: Supplementary file 1 — Supplementary Information. [file 41598_2021_82934_MOESM1_ESM.pdf]
